# The SASP factor IL‐6 sustains cell‐autonomous senescent cells via a cGAS‐STING‐NFκB intracrine senescent noncanonical pathway

**DOI:** 10.1111/acel.14258

**Published:** 2024-07-16

**Authors:** Florencia Herbstein, Melanie Sapochnik, Alejandra Attorresi, Cora Pollak, Sergio Senin, David Gonilski‐Pacin, Nicolas Ciancio del Giudice, Manuel Fiz, Belén Elguero, Mariana Fuertes, Lara Müller, Marily Theodoropoulou, Lucas B. Pontel, Eduardo Arzt

**Affiliations:** ^1^ Instituto de Investigación en Biomedicina de Buenos Aires (IBioBA)—CONICET—Partner Institute of the Max Planck Society Buenos Aires Argentina; ^2^ Departamento de Fisiología y Biología Molecular y Celular, Facultad de Ciencias Exactas y Naturales Universidad de Buenos Aires Buenos Aires Argentina; ^3^ Medizinische Klinik und Poliklinik IV Ludwig‐Maximilians‐Universität (LMU) München Munich Germany; ^4^ Present address: Josep Carreras Leukaemia Research Institute (IJC) Badalona Spain

**Keywords:** interleukin‐6, intracellular, pituitary, senescence, signaling, therapy‐induced senescence, tumors

## Abstract

Senescent cells produce a Senescence‐Associated Secretory Phenotype (SASP) that involves factors with diverse and sometimes contradictory activities. One key SASP factor, interleukin‐6 (IL‐6), has the potential to amplify cellular senescence in the SASP‐producing cells in an autocrine action, while simultaneously inducing proliferation in the neighboring cells. The underlying mechanisms for the contrasting actions remain unclear. We found that the senescence action does not involve IL‐6 secretion nor the interaction with the receptor expressed in the membrane but is amplified through an intracrine mechanism. IL‐6 sustains intracrine senescence interacting with the intracellular IL‐6 receptor located in anterograde traffic specialized structures, with cytosolic DNA, cGAS‐STING, and NFκB activation. This pathway triggered by intracellular IL‐6 significantly contributes to cell‐autonomous induction of senescence and impacts in tumor growth control. Inactivation of IL‐6 in somatotrophic senescent cells transforms them into strongly tumorigenic in NOD/SCID mice, while re‐expression of IL‐6 restores senescence control of tumor growth. The intracrine senescent IL‐6 pathway is further evidenced in three human cellular models of therapy‐induced senescence. The compartmentalization of the intracellular signaling, in contrast to the paracrine tumorigenic action, provides a pathway for IL‐6 to sustain cell‐autonomous senescent cells, driving the SASP, and opens new avenues for clinical consideration to senescence‐based therapies.

## Graphical abstract

Senescent cells produce a secretory phenotype (SASP) that acts on surrounding cells activating multiple responses. In senescent cells the SASP key factor IL‐6 also sustains and amplifies senescence interacting with the intracellular IL‐6R in anterograde traffic structures with cytosolic DNA and cGAS‐STING‐NFκB activation. In addition to the known autocrine and paracrine proliferative action of secreted IL‐6, not secreted IL‐6 sustains cell‐autonomous senescence through this intracrine pathway.

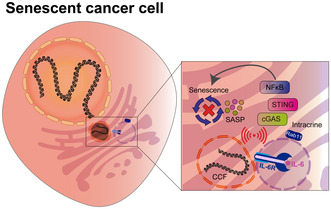

**Abbreviations**
AKT: serine/threonine kinase 1
BFA: Brefeldin A
CCF: cytosolic chromatin fragments
cGAS: cyclic GMP‐AMP synthase
CHOP: C/EBP homologous protein
CRISPR: Clustered Regularly Interspaced Short Palindromic Repeats
DAPI: 4′,6‐diamidino‐2‐phenylindole
Dox: doxorubicin
ER: endoplasmic reticulum
EV: extracellular vesicles
FS: folliculostellate cells
GFP: green fluorescent protein
GH: growth hormone
gp130: glycoprotein 130
IFN: interferon
IL‐1 β: interleukin‐1β
IL‐2: interleukin‐2
IL‐6: interleukin‐6
IL‐6R: interleukin‐6 receptor
IκBα: nuclear factor‐kappa B inhibitor alpha
MSC: mesenchymal stromal cells
NFκB: nuclear factor kappa‐light‐chain‐enhancer of activated B cells
NOD/SCID: nonobese diabetic/severe combined immunodeficiency
OIS: oncogene‐induced senescence
p38MAPKs: p38 mitogen‐activated protein kinases
PI3K: phosphoinositide 3‐kinases
RFP: red fluorescent protein
SASP: senescence associated secretory phenotype
SA‐β‐gal: senescence‐associated β galactosidase
scr: scrambled
siRNA: small interfering RNA
STAT: signal transducer and activator of transcription
STING: stimulator of interferon genes
TAF: tumor associated‐fibroblasts
TBK1: TANK‐binding kinase 1
TIS: therapy‐induced senescence
TZ: tocilizumab
UPR: unfolded protein response
WB: western blot

## 1 INTRODUCTION

Cellular senescence, a complex and diverse response characterized by an interruption in cell replication and maintenance of cellular viability, has been widely reported in both physiological and pathological contexts and as key component in age‐related diseases and cancer (Lopez‐Otin et al., 2023; Yousefzadeh et al., 2021). Senescent cells are metabolically active and experience changes in the expression and secretion of proteins with complex and not yet fully understood activities, collectively termed as senescence‐associated secretory phenotype (SASP) (Birch & Gil, 2020). The SASP includes several families of soluble factors that act on the surrounding cells with pro‐senescence activity and by activating several cell surface receptors and the corresponding signal transduction pathways leading to multiple responses, frequently associated with the development of pathologies (Birch & Gil, 2020).

Oncogene‐induced senescence (OIS), a particular type of senescence triggered by oncogene activation in the cell, was described in different premalignant lesions in vitro and in vivo (Collado et al., 2005). It is specifically linked to an inflammatory transcriptome, which includes the pleiotropic cytokine interleukin‐6 (IL‐6) (Coppe et al., 2010). IL‐6 and other SASP factors promote tumorigenesis and cell proliferation, but they can also exert tumor suppressor functions and trigger an immune response, favoring the reduction of tumoral cells and the regression of cancer.

Pituitary tumors account for 15% of all intracranial neoplasms in adults and rarely undergo malignant transformation (Melmed, 2011). OIS has been postulated to explain this unique feature. In pituitary tumors, and in particular the growth hormone (GH)‐secreting ones, SA‐β‐Gal abundance is increased compared with normal pituitary tissue (Manojlovic‐Gacic et al., 2016). In addition, GH was recently proposed as a nonconventional SASP factor that accumulates intracellularly in somatotroph tumors and activates DNA damage response triggering senescence (Chesnokova & Melmed, 2022).

In the pituitary IL‐6 has a dual action. In the normal gland, IL‐6 is secreted by folliculostellate cells (FS), that surround hormone‐secreting cells and acts in a paracrine manner to stimulate pituitary cell growth (Graciarena et al., 2004). In addition, it stimulates the secretion by FS of vascular endothelial growth factor and the matrix metalloproteases, thereby triggering the formation of vessels and remodeling of the extracellular matrix. IL‐6 is also synthesized and secreted directly by pituitary tumor cells, which also express its receptor, IL‐6R (Jones et al., 1994). IL‐6 also participates in the senescence‐mediated paracrine oncogenesis in aggressive pediatric pituitary tumors (Gonzalez‐Meljem & Martinez‐Barbera, 2021).

In an experimental model of melanoma cells IL‐6 was shown to have a senescence‐promoting action via an autocrine loop and to induce cell proliferation via its paracrine action (Kuilman et al., 2008). In the pituitary, IL‐6 produced by FS cells acts in paracrine fashion to trigger proliferation in pituitary tumor cell models, including MtT/S cells, which in the tumoral stage produce IL‐6 that induces autocrine senescence (Sapochnik et al., 2017). IL‐6 was reported to have senescent and anti‐senescent actions in radiation‐induced senescence in salivary glands, indicating two independent levels of action.

An intriguing question is how cells discriminate these signals. Does IL‐6 secreted by the senescent tumor cells act by binding to its membrane receptor in an autocrine loop? Or does it trigger senescence without being secreted, acting in an intracrine fashion? The aim of this work, using different cellular models, the somatotroph tumor cell line MtT/S as a natural senescent model (Sapochnik et al., 2017) and glioblastoma, pulmonary, and melanoma senescent cells, is to establish the mechanism that mediates this senescent‐inducing action of IL‐6, in order to delineate the pathways that control cellular senescence.

## 2 MATERIALS AND METHODS

### 2.1 Cell culture and treatments

MtT/S cells (Cat# RCB0528, RRID:CVCL_H713), from a rat somatotrophic pituitary cell line obtained from an estrogen‐induced somatotrophic tumor, were cultured as described (Graciarena et al., 2004). These cells were directly obtained from K. Inoue (Gunma University, Japan) who generated the cells. A549, a non‐senescent adenocarcinoma cell line (RRID:CVCL_0023) and U87MG glioblastoma cell line (RRID:CVCL_0022), obtained from the American Type Culture Collection (ATCC) were kindly provided by C. Perez Castro (CONICET, Argentina). A375 melanoma cell line obtained from the ATCC was kindly provided by G. Rabinovich (CONICET, Argentina). Cells were regularly tested for Mycoplasma.

For intracellular protein traffic disruption, cells were treated with 100 ng/mL BFA (Sigma Aldrich‐B7651 in ethanol, St. Louis, MO, USA) diluted in 10% FBS DMEM. For inducing senescence in A549 cells Doxorubicin hydrochloride (IMA Laboratory, Argentina) 132 nM was used for 48 h diluted in 10% FBS DMEM. IL‐1β (R&D‐401‐ML‐005 in BSA‐PBS, Minneapolis, USA) was used in A549 cells at a concentration of 20 ng/mL for 24 h. To trigger senescence Dox 100 nM was used for 48 h diluted in 10% FBS DMEM in U87MG cells and Cisplatin (sc‐200896 Santa Cruz Biotechnologies, Dallas, TX, USA) 2 μM for 24 h in A375 cells. Tocilizumab/Actemra (Roche Molecular Biochemicals, Argentina) was used at the following concentrations and times: A549 (48 h; 100 ng/mL), A375 (72 h; 100 μg/mL), and U87MG (48 h; 10 μg/mL). Human IL6R neutralizing antibody was used in 10% FBS DMEM at a concentration of 10 μg/mL for 48 h (R&D AB‐227‐NA in PBS).

Dyngo‐4a (30 μm) (Abcam‐ab120689 in DMSO, Cambridge, United Kingdom) was used in Opti‐MEM. CHX (100 μg/mL) (Sigma Aldrich‐01810, in DMSO) was used in 10% FBS. For NFκB and p38MAPK inhibition, sulfasalazine (3 mM) (Sigma Aldrich‐S0883), and SB203580 (SB20) (5 μM) (Calbiochem 559389 in DMSO, Darmstadt, Germany) were used in 10% FBS DMEM or Opti‐MEM, respectively. For the pharmacological inhibition of STING, H‐151 (Invivogen, inh‐h151 in DMSO, Toulouse, France) was used at a concentration of 5 μM for 5 h Recombinant human IL‐6 (rhIL‐6) (R&D 206‐IL‐010 in BSA‐PBS) was used to stimulate the JAK/STAT3 pathway at a concentration of 50 ng/mL for 30 min. Control experiments were performed in the presence of an equivalent amount of the respective drug dilution buffer.

For all experiments, unless otherwise stated, reagents were obtained from Thermo Fisher Scientific (Waltham, MA, USA) or Sigma Aldrich.

### 2.2 Senescence biomarkers

To characterize senescence cells, we followed the proposed multi‐marker approach (Gorgoulis et al., 2019), evaluating SA‐β‐Gal activity, expression of p16, p21, pRb, lamin B1, IL‐6 (component of the SASP), and cell phenotype.

### 2.3 SA‐β‐gal activity

Cytochemical identification of SA‐β‐gal positive cells was carried out as outlined (Debacq‐Chainiaux et al., 2009) based on the development of a blue color after the exposure of fixed cells to the chromogenic substrate of β‐gal and X‐Gal. Cells were incubated between 12 and 16 h in a CO_2_‐free incubator and examined by a bright field microscopy.

Fluorescence detection of SA‐β‐gal enzymatic activity was performed as described (Debacq‐Chainiaux et al., 2009) using flow cytometry (BD Becton Dickinson, FACS CantoII, NJ, USA). SA‐β‐gal activity was measured by the mean fluorescence intensity (MFI) using C_12_FDG as a substrate that becomes fluorescent after cleavage by the enzyme. Only senescent cells are detected at pH 6.0.

### 2.4 Plasmids and transfections

MtT/S and A549 cells transient transfection was performed for 48 h of expression using Lipofectamine 2000 reagent following the manufacturer's instructions, with the following plasmids: IL‐6‐GFP (RRID:Addgene_28088) (Addgene, Cambridge, MA, USA), IL‐6R‐RFP (MG50280‐ACR, Sino Biological, Shanghai, China), IκB plasmid coding for an IκB mutated at Ser32 and Ser36 that cannot be phosphorylated and degraded, kindly provided by Dr. B. Kaltschmidt (Univ. Freiburg, Germany) and NFκB‐LUC construct, with three NFκB binding sites from the human immunodeficiency virus cloned upstream of the luciferase gene, kindly provided by Dr. M. Bell (Mayo Clinic, Rochester, MN, USA). Because of the known directly regulatory interplay of NFκB and Rb phosphorylation (Jin et al., 2019), pRb has not been used as a marker of the senescence state of the cells under NFκB direct modulation. Rab11‐DN‐EGFP plasmid was kindly provided by Dr. Francisca Bronfman (Andres Bello University, Chile). IL‐6‐GFP‐KDEL plasmid was generously provided by Dr. Ying Zhu (Wuhan University, China) and IL‐6 and IL‐6‐KDEL plasmids were kindly provided by Dr. Stefan Rose‐John (Christian‐Albrechts‐University, Germany). STING expression was knocked down by siRNA transfection targeting mouse and human protein (Sigma Aldrich‐EMU074691, EHU133481) for MtT/S and A549 cells, respectively.

### 2.5 Western immunoblot

WB analysis was performed as previously described (Sapochnik et al., 2017). Cells lysates were prepared in 2× Laemmly buffer and separated in sodium dodecyl sulfate polyacrylamide gel electrophoresis (SDS‐PAGE). Membranes were incubated with specific primary antibodies, followed by incubation with HRP‐conjugated secondary antibodies (Bio‐Rad Laboratories, Hercules, CA, USA). Developing was performed with the Super Signal West Dura kit according to manufacturer's instruction (Pierce Biotechnology, Waltham, MA, USA) using G:BOX‐CHEMI‐XT4 (Synoptics Ltd., Cambridge, United Kingdom). The following antibodies were used: p16 (Cat# sc‐1661, RRID:AB_628067) (1:1000), STAT3 (Cat# sc‐8019, RRID:AB_628293) (1:1000), p‐STAT3 (Cat# sc‐8059, RRID:AB_628292) (1:1000), β‐Actin (Cat# sc‐47,778, RRID:AB_626632) (1:3000) (Santa Cruz Biotechnologies, Dallas, TX, USA), pRb (Cat# 9307, RRID:AB_330015) (1:1000), NFκB (Cat# 8242, RRID:AB_10859369) (1:1000), p‐p65 (Cat# 3033, RRID:AB_331284) (1:1000), p38 (Cat# 9212, RRID:AB_330713) (1:1000), p‐p38 (Cat# 9216, RRID:AB_331296) (1:1000), Akt (Cat# 2920, RRID:AB_1147620) (1:1000), p‐Akt (Cat# 4058, RRID:AB_331168) (1:1000), p21 (Cat# 2947, RRID:AB_823586) (1:1000), TBK1 (Cat# 3504T, RRID:AB_2255663) (1:1000), pTBK1 (Cat# 5483 T, RRID:AB_10693472) (1:1000) (Cell Signaling Technology, Danvers, MA, USA), GAPDH (Cat# ab8245, RRID:AB_2107448) (1:10000), IL‐6 (Cat# ab9324, RRID:AB_307175) (1:500) (Abcam), STING (Cat# NBP2‐24683, RRID:AB_2868483) (1:1000) (Novus Biologicals, Colorado, USA), and pSTING (Cat# PA5‐105674, RRID:AB_2817102) (1:1000) Thermo Fisher. Western immunoblot analyses were quantified with Fiji‐ImageJ software. Actin or GAPDH were used as loading controls for pRb since total Rb levels are influenced by the cell cycle, which makes it difficult to standardize its levels as a loading control (Jacobberger et al., 2008; Whittaker et al., 2004).

### 2.6 Immunofluorescence and image analysis

MtT/S cells were fixed in 4% paraformaldehyde and blocked in 1% bovine serum albumin (1 h, room temperature), after permeabilization with 0.1% (v/v) triton X‐100. Cells were incubated (4°C, 16 h) with antibodies against, Sec31A (Cat# 13466, RRID:AB_2798228), GOPC (Cat# 8576, RRID:AB_10891809), Syntaxin (Cat# 2869, RRID:AB_2196500), cGAS (Cat# 79978, RRID:AB_2905508), Rab11 (Cat# 5589, RRID:AB_10693925), CHOP (Cat# 2895 T RRID:AB_2089254), GRASP65 (Cat# PA3‐910, RRID:AB_2113207), cGAS (Cat# PA5‐121188, RRID:AB_2914760) (1:100, Thermo Fisher), STING (5 μg/mL, Novus Biologicals), STING (1:50, Abcam), Lamin B1 (ab16048, RRID:AB_443298) (1:1000, Abcam), and IL6R (15 μg/mL, R&D). After washing, the corresponding Alexa Fluor® 647 (Cat# A‐21235, RRID:AB_2535804) or 555 anti‐rabbit and anti‐mouse secondary antibody (Cat# A‐21422, RRID:AB_2535844) (1:500, Thermo Fisher) was added (1 h, room temperature). Nuclei were stained with 4′,6‐diamidino‐2‐phenylindole (DAPI). Cells were mounted with Mowiol mounting medium (Sigma Aldrich), and images were acquired in a LSM 710 AxioObserver confocal microscope (RRID:SCR_018163, Carl ZEISS, Oberkochen, Germany). Images were acquired with ZEN 2011 software (Carl‐Zeiss) and analyzed with Fiji‐ImageJ software (RRID:SCR_003070). Scatter plots and co‐ocurrence pixel maps were obtained with ImageJ Colocalization threshold plugin.

### 2.7 Luciferase assay

Cells were transfected with NFκB‐LUC and CMV‐β‐gal to standardize the results. After 24 h, cells were washed with PBS and lysed with Passive Lysis Buffer (Promega, Madison, WI, USA). Luciferase activity was measured using a Luciferase detection kit (Promega) with a Junior Luminometer (Berlthod, Bad Wildbad, Germany).

For β‐galactosidase activity, 100 μL of β‐galactosidase buffer (100 mM Na2HPO4/NaH2PO4 pH 7.4, 1 mM MgCl2, 50 mM β‐mercaptoethanol, and 0.66 mg/mL 2‐Nitrophenyl β‐D‐galactopyranoside (ONPG)) were added to 20 μL of lysates. After incubation at 37°C, β‐galactosidase activity was determined using iMarkTM Microplate Reader (Bio‐Rad) at 415 nm.

### 2.8 Cell viability and proliferation

1 × 10^3^ cells were plated in 96‐well plates and 72 h later a WST‐1 (Roche Molecular Biochemicals, Basel, Switzerland) assay or 44 mM resazurin sodium salt (Sigma Aldrich) was added to each well and was used to measure proliferation following the manufacturer's instructions. The reaction products were measured in an ELISA plate at 450 nm or a florescent plate reader with excitation at 530 nm and emission at 590 nm, respectively.

Trypan blue exclusion staining was used for viability assessment.

### 2.9 MtT/S IL‐6 deficient cells by CRISPR/Cas9

Guide sequences targeting exon 2 of rat IL‐6 were design using the Benchling software (RRID:SCR_013955) and cloned into pX458 vector (RRID:Addgene_48138). Guide sequences can be found in Table S1. MtT/S cells were transfected with the CRISPR plasmid using Lipofectamine 2000 according to the manufacturer's protocol. Two days post‐transfection, GFP‐positive cells were sorted and plated using BD FACSAria Fusion cell sorter. After three rounds of transfection‐sorting, single cells were plated into 96‐well plate (Figure S6). Cells were plated at 80% confluence and 24 h late medium was collected and stocked at −80°C until IL‐6 determination. Protein knock out was confirmed with an IL‐6 ELISA Kit (R6000B, BD Becton Dickinson). Genomic DNA was extracted from individual clones and targeted locus was PCR amplified and subjected to Sanger sequencing. The primers used to amplify the targeted locus by PCR are shown in Table S1.

### 2.10 Animal experiments

MtT/S cells (MtT/S IL‐6 deficient clones and MtT/S WT) were harvested by trypsinization, washed twice with PBS, resuspended in DMEM, and injected subcutaneously, as described (Graciarena et al., 2004), into the flanks of 6‐ to 8‐week‐old male NOD/SCID mice (RRID:IMSR_JAX:001,303). Animals were housed with access to food and water ad libitum in ventilated mouse cages (1–5 mice per cage) at the Institute Animal Services Facility. All animal experimental protocols were approved by the Ethical Committee on Animal Care and Use (CICUAL), University of Buenos Aires, Argentina and performed in compliance with ARRIVE Animal Research guidelines recommendations. Four independent groups of four mice were injected with 5 × 10^5^ cells (WT cells, #58 and #66 IL‐6 knock out clones and IL‐6 knockdown clone called Intermediate). Animals were examined for tumor formation every 3 days and tumor growth was determined as described (Graciarena et al., 2004).

### 2.11 Statistical analysis

Prism software version 6.0a (GraphPad Software, Inc, RRID:SCR_002798) was used for all statistical analyses. To determine statistical significance between groups, Student's *t* test (unpaired, two‐tailed; for two groups) and one‐way analysis of variance (ANOVA; for three or more groups) with post hoc Tukey's or Dunnett's test, were used assuming normal distribution of the data.

Statistical significance was accepted at *p* < 0.05. Data are presented as mean ± standard error of the mean (SEM), *n* refers to biological replicates. In all cases replicate experiments were performed independenty.

## 3 RESULTS

### 3.1 IL‐6 induces senescence independently of its cell membrane receptor and endocytosis

Considering that IL‐6 is secreted via the classical secretory pathway and transit recycling endosomes (Manderson et al., 2007) we first manipulated ER‐Golgi transit and vesicular trafficking to generate cytokine accumulation. To determine whether autocrine IL‐6 utilizes its membrane receptor (requiring secretion from the cell) or functions from within the cell itself in an intracrine fashion, we initially employed pharmacological inhibition, treating rat somatotrophic MtT/S cells with brefeldin A (BFA) that inhibits vesicle assembly in the Golgi and subsequently protein transport and secretion. It has been shown that at the time—5 h—and doses used, cellular processes such as senescence, autophagy, apoptosis, or endoplasmic reticulum (ER) stress are not affected by BFA treatment (Narita et al., 2011; Verboogen et al., 2018), in contrast to a long—72 h—treatment with BFA (McHugh et al., 2023).

**FIGURE 1:**
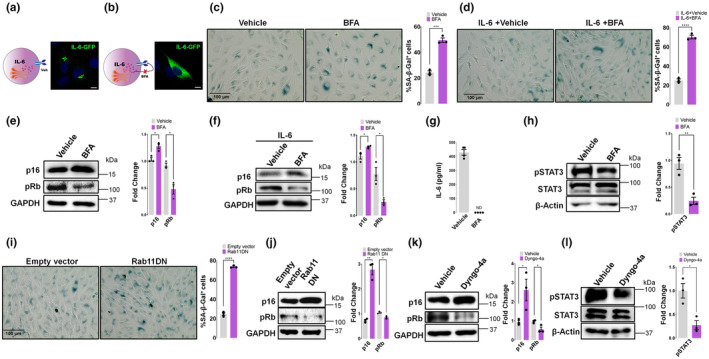
IL‐6 intracellular accumulation increases senescence markers in pituitary tumor cells (a, b) Scheme representing the traffic of IL‐6 and representative confocal images of MtT/S cells transiently transfected with an expression vector of IL‐6 fused to GFP (IL‐6‐GFP) (green) and treated with Brefeldin A 100 ng/mL, 5 h (BFA) or ethanol, 5 h (vehicle). DAPI (blue) was used to stain cell nuclei. (c, d) Representative brightfield images of Senescence associated‐β‐galactosidase (SA‐β‐gal) staining after 5 h of treatment with BFA or vehicle under basal condition (c) or IL‐6 transient overexpression (d). The percentage of cell staining positive for SA‐β‐gal is shown. Bars show the mean ± SEM of three independent experiments (dots). (e, f) Immunoblots of p16 and pRb of MtT/S cells extracts under basal condition (e) or IL‐6‐overexpression (f) after 5 h treatment with BFA or vehicle. (g) IL‐6 levels were determined by ELISA assay after 5 h treatment with BFA or vehicle under IL‐6 transient overexpression conditions in the secreted media of MtT/S cells. Data in the bar chart show mean ± SEM of quadruplicates of one of two experiments (*n* = 2) with similar results. ND: not detected. (h) Immunoblots of pSTAT3 and STAT3 after treatment with BFA or vehicle. (i) Representative brightfield images of Senescence associated‐β‐galactosidase (SA‐β‐gal) staining of MtT/S cells after expression of a dominant negative form of Rab11 or empty vector. The percentage of cell staining positive for SA‐β‐gal is shown. Bars show the mean ± SEM of three independent experiments (dots). (j) Immunoblot of p16 and pRb after expression of a dominant negative form of Rab11 or empty vector. (k, l) Immunoblot of p16 and pRb (k) or pSTAT3 (l) after 5 h treatment with Dyngo‐4a (30 μM) or vehicle (DMSO). Blots are representatives of three independent experiments with similar results. GAPDH or β‐Actin were used as a loading control. Bars show mean ± SEM quantification of independent (dots) immunoblots relative to vehicle‐treated samples (e, f, h, k, l). Quantification of pSTAT3 shows the ratio of pSTAT3/STAT3 (h, l). Scale bar, 100 μm (c, d, i). ****p < 0.0001, ***p < 0.001, **p < 0.01 *p < 0.05, ns, not significant. *p* value was calculated using an unpaired Student's *t* test.

BFA inhibition of the secretory pathway increased intracellular IL‐6 levels (Figure 1a versus Figure 1b). Under both basal and IL‐6 overexpression conditions, BFA treatment augmented the senescence‐associated beta‐galactosidase (SA‐β‐Gal) positive cells (Figure 1c,d and Figure S1a,b), and p16 levels, while concurrently reducing Rb phosphorylation (Figure 1e,f). Under IL‐6 overexpression, after BFA treatment, IL‐6 was not detected in the secreted media of the cells while, in contrast, in vehicle conditions IL‐6 was secreted (Figure 1g), indicating limited accumulation after the transient transfection without BFA.

Inhibition of IL‐6 secretion, in contrast to the stimulatory action of IL‐6 on the membrane receptor (Sapochnik et al., 2017) decreased downstream STAT3 phosphorylation levels (Figure 1h). BFA did not affect cell viability, was reversible and did not alter the C/EBP homologous protein (CHOP) ER stress marker (Figure S1c–g).

In addition, using an independent experimental approach, overexpression of a dominant negative mutant form of Rab11 in MtT/S cells, which blocks IL‐6 endosomal transport and secretion (Manderson et al., 2007) had similar effects on senescence biomarkers (Figure 1i,j and Figure S1h).

Exposing cells to Dyngo‐4a, which blocks the endocytosis of the IL‐6 bounded IL‐6R and the signaling from early endosomes (Shah et al., 2002), did not produce changes that reverse the pattern in senescence induction, with p16 levels being induced and Rb phosphorylation decreased (Figure 1k); STAT3 phosphorylation decreased as expected (Figure 1l). These data confirm that IL‐6‐induced senescence was triggered independently of the endocytosis of membrane receptor and indicate that endocytosis of the IL‐6/IL‐6R complex is not involved in the activation of the senescence mechanism.

Altogether, these results indicate that in MtT/S cells, IL‐6 induces senescence biomarkers from inside the cell and does not involve the binding to the receptor expressed in the membrane and endocytosis.

### 3.2 Subcellular localization of IL‐6 and its receptor in structures involved in anterograde transport in MtT/S cells

**FIGURE 2:**
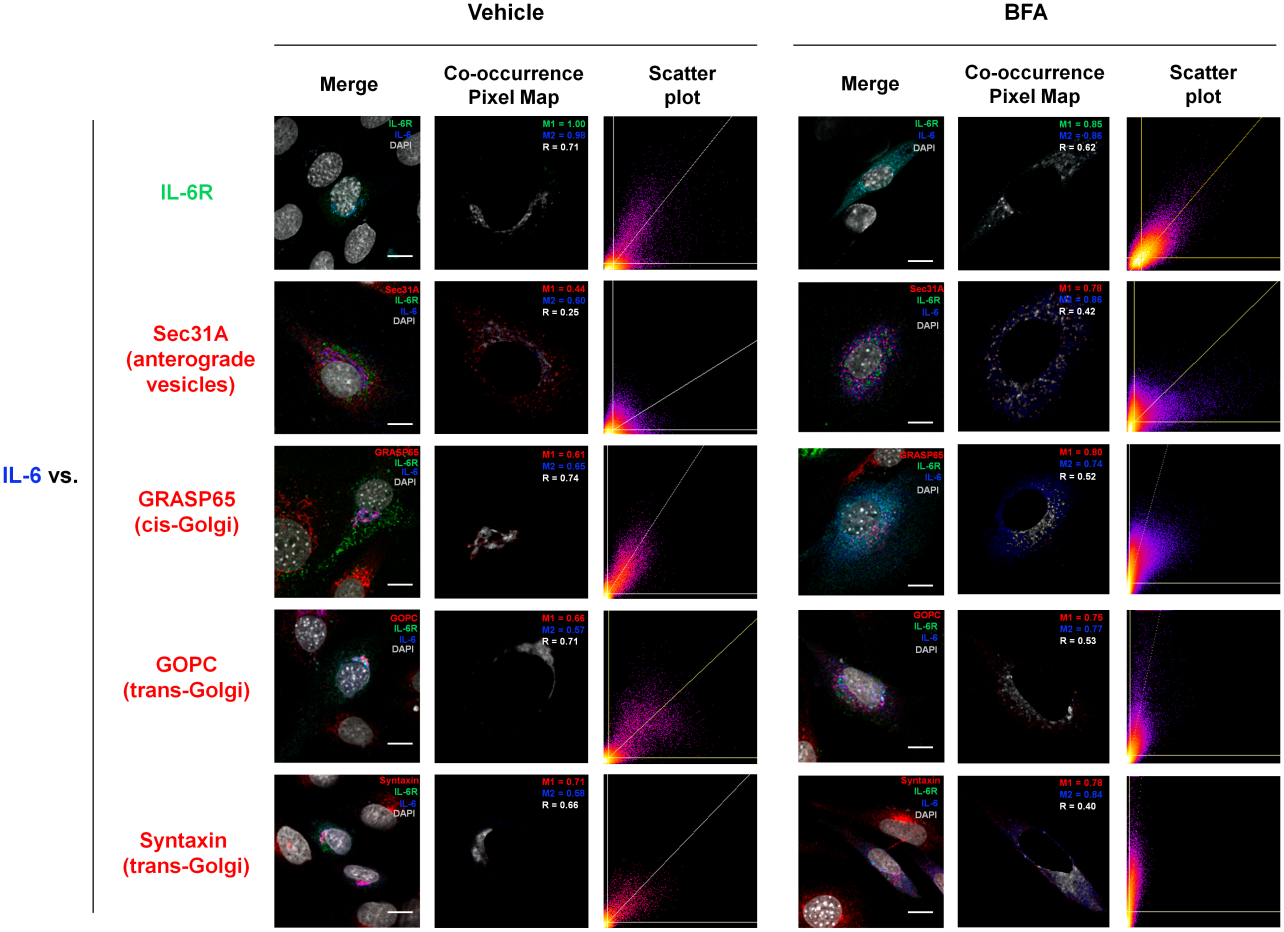
IL‐6 and IL‐6R localization within the cell and in anterograde structures. Representative merged immunofluorescent confocal images (first and fourth columns) using a specific antibody against Sec31A, GRASP65, GOPC, or Syntaxin (red), of MtT/S cells co‐transfected with IL‐6‐GFP (blue) and IL‐6R‐RFP (green). Cells were treated with Brefeldin A (100 ng/mL) (right panel) or vehicle (ethanol) (5 h) (left panel). DAPI (gray) was used to stain cell nuclei. Scale bars = 10 μm. Images are representatives of three experiments with similar results. Co‐occurrence Pixel Maps show co‐localized pixels in grayscale and Scatter Plots show the correlation of pixels in a multicolor palette that indicate events density for IL‐6 versus IL‐6R‐RFP or Sec31A, GRASP65, GOPC, or Syntaxin in cell cytoplasm, excluding the nuclei, for each condition. Manders coefficients for each channel (M1 and M2) and Pearson's correlation coefficient (R) are shown.

A necessary condition for intracellular signaling is that IL‐6 is associated with its receptor in subcellular compartments from which they may signal. To monitor the subcellular localization of IL‐6 and IL‐6R we overexpressed in MtT/S cells plasmids coding for IL‐6 and IL‐6R fused to GFP and RFP, respectively, (IL‐6‐GFP; IL‐6R‐RFP) which show distribution coincidence with and without BFA treatment (Figure 2).

IL6‐GFP and IL‐6R‐RFP intracellular localizations overlapped with compartments positive for anterograde vesicles protein Sec31A, cis‐Golgi marker GRASP65, trans‐Golgi markers GOPC, and Syntaxin (Figure 2). BFA's action on disrupting the Golgi was verified with GRASP65, GOPC, and Syntaxin (Figure 2).

These results support the notion that receptor bound IL‐6 may activate senescent signals during anterograde transport.

### 3.3 cGAS‐STING signaling pathway is implicated in the intracellular IL‐6/IL‐6R spatial trafficking and signaling in naturally senescent pituitary tumor cells

**FIGURE 3:**
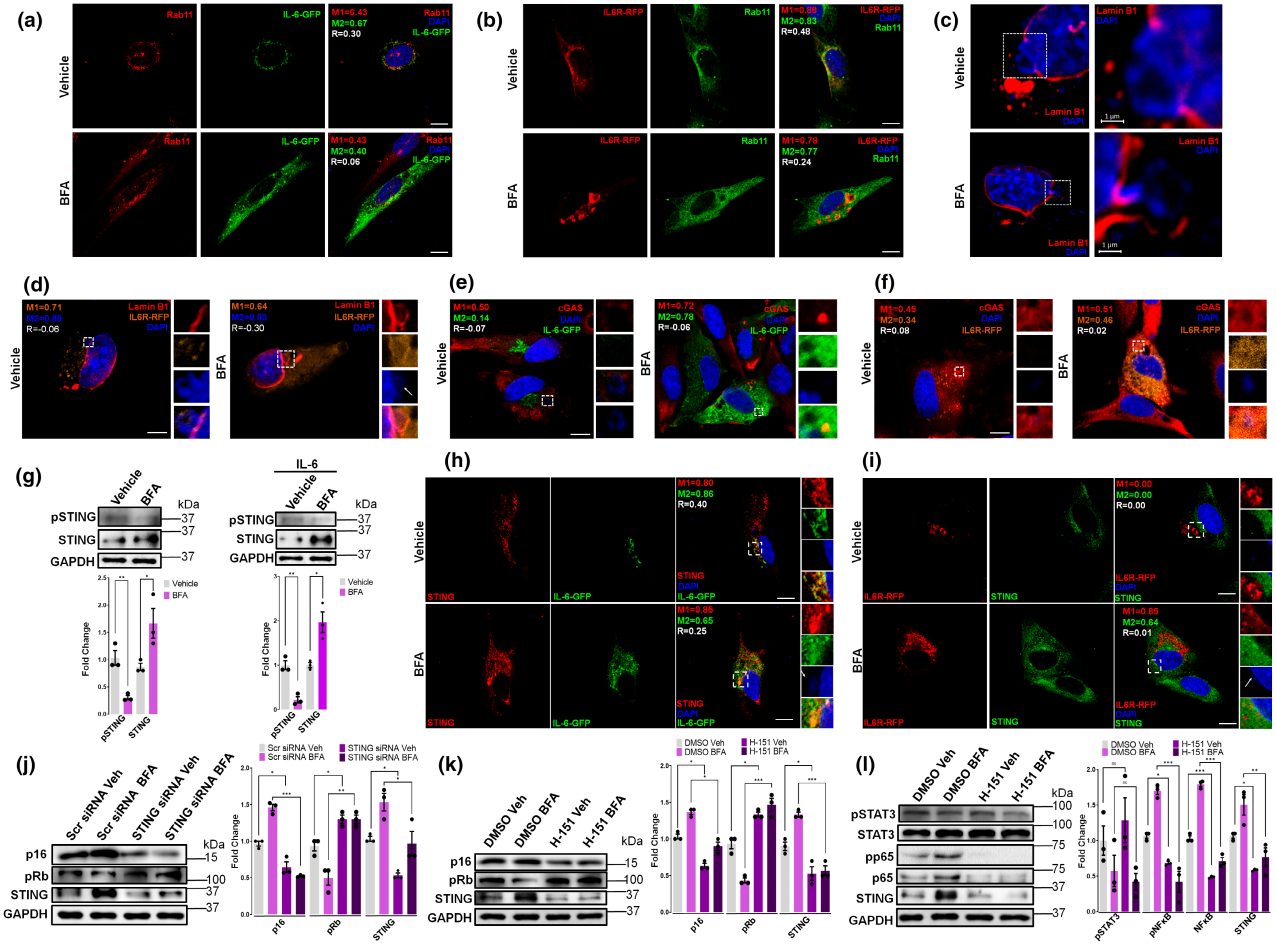
cGAS‐STING pathway signals IL‐6 induced senescence activated by chromatin fragments in naturally senescent pituitary tumor cells. (a–f, h, i) Representative immunofluorescent confocal images using a specific antibody against Rab11, lamin B1, cGAS, or STING of MtT/S whether transfected (a, b, d, e, f, h, i) or not (c) with IL‐6‐GFP or IL‐6R‐RFP treated with Brefeldin A 100 ng/mL, 5 h (BFA) or ethanol, 5 h (vehicle). DAPI (blue) was used to stain cell nuclei. DAPI‐stained fragments are indicated by white arrows. Manders coefficients for each channel (M1 and M2) and Pearson's correlation coefficient (R) were calculated in cell cytoplasm (a, b), excluding the nuclei, for each condition (8–12 cells per experiment) and in zoomed views of selected regions of interest (c–f, h and i). Particular focus is made on the confocal images illustrating the loss of lamin B1 that impacts nuclear envelope's integrity (Figure 3c, d). Images are representatives of three experiments with similar results. Scale bars = 10 μm. (g) Immunoblots of STING and pSTING of MtT/S cells extracts under basal condition (left) and IL‐6‐overexpression (right) after 5 h treatment with BFA or vehicle. Quantification of pSTING shows the ratio of pSTING/STING. (j–l) Immunoblots of p16, pRb, STING, pSTAT3, STAT3, p65, and pp65 of MtT/S cells extracts treated with scramble or STING siRNA, STING antagonist H‐151 (5 μM), or vehicle (DMSO) for 5 h in combination with BFA or its vehicle. *p* value was calculated using ANOVA, followed by Tukey test. Blots are representatives of three independent experiments with similar results. Quantification of pSTAT3 shows the ratio of pSTAT3/STAT3 and for pp65 the ratio of pp65/p65 (l). GAPDH was used as a loading control. Bars show mean ± SEM quantification of independent (dots) immunoblots relative to vehicle‐treated samples (g, j–l). ***p < 0.001, **p < 0.01, *p < 0.05, ns, not significant. *p* value was calculated using an unpaired Student's *t* test or where indicated, ANOVA.

Following the observation that inhibiting Rab11‐mediated trafficking increased IL‐6's impact on senescence‐related markers (Figure 1i,j), we observed IL‐6 and IL‐6R colocalization with Rab11, even under BFA treatment that raises IL‐6 intracellular levels, suggesting the involvement of the recycling endosomes in IL‐6 increase (Figure 3a,b).

We detected lamin B1 labelled nuclear envelope protrusion and loss of nuclear lamina integrity, which is a senescent‐reported feature, in both BFA and non‐BFA MtT/S treated cells (Figure 3c). Furthermore, we observed DAPI‐stained DNA fragments in the perinuclear regions bearing IL‐6/IL‐6R signals, which led us to investigate the participation of the cGAS‐STING pathway that associates with cytosolic DNA sensing. In areas where the chromatin fragments are observed, we noticed the presence of cGAS, IL‐6, or IL‐6R in MtT/S cells (Figure 3d–f).

BFA treatment increased STING expression levels, but it decreased its phosphorylation levels (Figure 3g). Furthermore, STING and IL‐6 were adjacent under both basal and BFA treatment, but BFA‐treated cells presented with a more diffuse distribution of STING (Figure 3h). We observed the same pattern for IL‐6R distribution after BFA exposure (Figure 3i). Knocking down STING with a siRNA (compared to the corresponding Scr control) or inhibiting with the pharmacological antagonist H‐151 (compared to the corresponding DMSO control), abolished the effects of BFA on senescent biomarkers and STING downstream mediator TANK‐binding kinase 1 protein (TBK1) (Figure 3j,k and Figure S2a). Unsurprisingly, this did not modify STAT3 phosphorylation levels but decreased NFκB phosphorylation levels (Figure 3l) prompting us to study NFκB as a mediator of senescence response downstream to STING activation.

IL‐6 activated NFκB in basal and BFA treated MtT/S cells (Figure S2b), confirming that it is part of its intracrine action. Overexpression of IκBα or treatment with the NFκB inhibitor sulfasalazine, both of which inhibited NFκB without affecting cell viability (Figure S2c–f), decreased senescent markers in both basal and BFA‐treated cells (Figure S2g,h), showing that the intracellular action of IL‐6 promotes senescence by activating NFκB. To study what mediates the stimulatory action of intracellular IL‐6 on NFκB activation, we examined members of the PI3K/Akt and p38MAPK pathways, which are known to activate NFκB (Xu et al., 2014). IL‐6 did not affect Akt phosphorylation upon BFA treatment (Figure S2i), suggesting that the PI3K/Akt pathway is not activated by intracellular IL‐6. Inhibiting p38MAPK with SB203580, a specific inhibitor of the p38MAPK pathway, reduced the phosphorylation of p38MAPK and of NFκB and decreased senescence markers (Figure S2j), indicating that IL‐6 intracellular action is mediated via the NFκB/p38MAPK pathway.

### 3.4 IL‐6 knock out cells form tumors in NOD/SCID mice and senescence is reestablished upon IL‐6 reexpression

**FIGURE 4:**
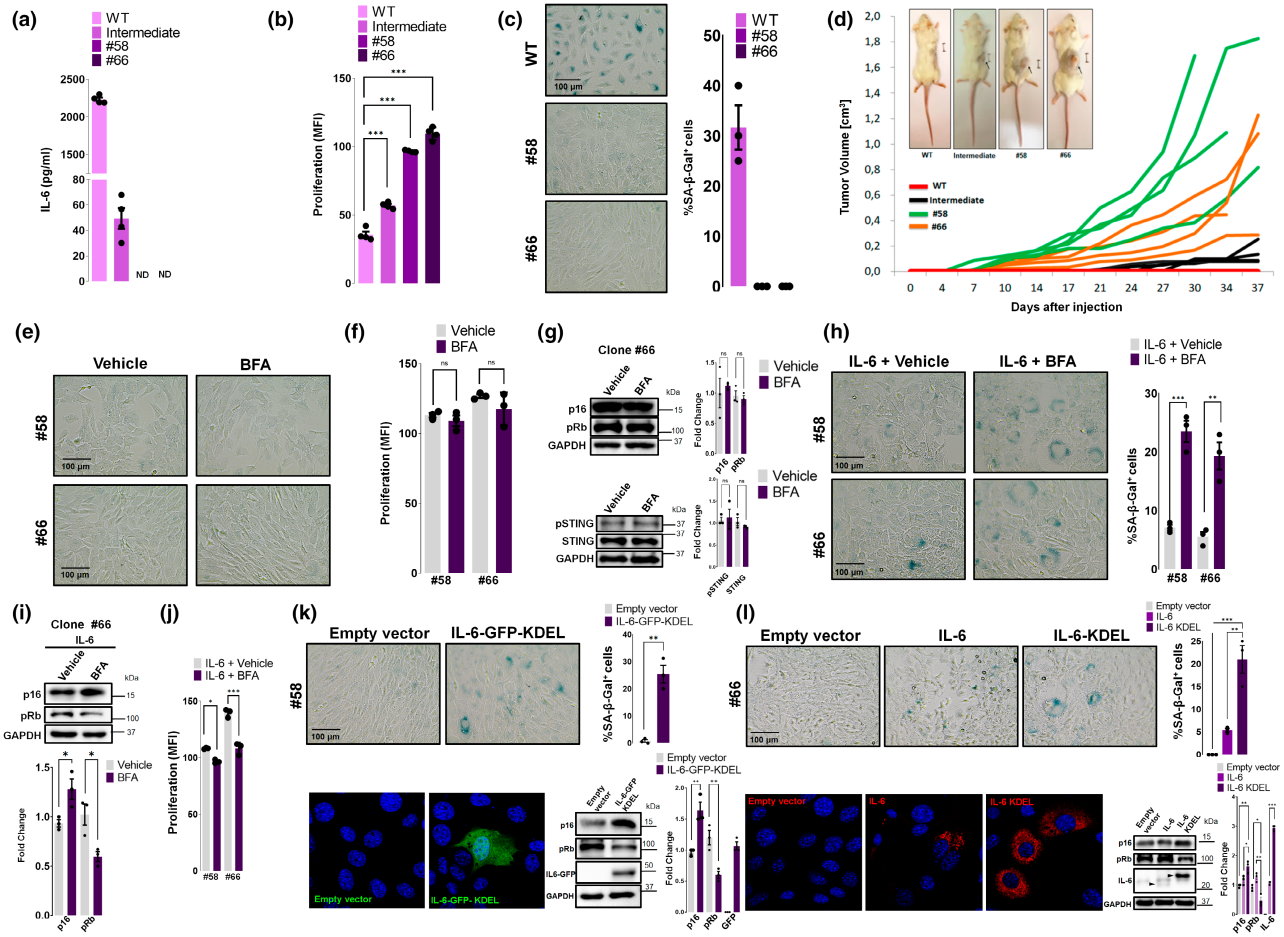
Inhibition of the secretory pathway increases senescence markers only when IL‐6 expression is reestablished in IL‐6 knock out MtT/S cells. (a) IL‐6 levels of wild type (WT) MtT/S cells and IL‐6 knock out (KO) clones (#58, #66 and Intermediate) were determined by ELISA after 24 h in culture. Data in the bar chart shows mean ± SEM of quadruplicates of one of two experiments (*n* = 2) with similar results. ND: not detected. (b) Cell proliferation was measured by resazurin reduction assay on WT MtT/S cells and IL‐6 KO clones. Dots in the bar chart show Mean Fluorescence Intensity (MFI) ± SEM of triplicates of one of three experiments (*n* = 3) with similar results. *p* value was calculated using ANOVA followed by Dunnett's multiple comparisons post test. (c) Representative brightfield images of SA‐β‐gal staining of wild type MtT/S and IL‐6 KO clones #58 and #66. The percentage of cell staining positive for SA‐β‐gal is shown. Bars show the mean ± SEM of three independent experiments (dots). (d) MtT/S WT cells and MtT/S IL‐6 KO clones were injected subcutaneously into NOD/SCID mice. Volume of each of the tumors with four mice in each group is shown. Photographs were taken at 30 days postinjection. One representative image of each group with similar results is shown. (e, h) Representative brightfield images of SA‐β‐gal staining of IL‐6 KO clones #58 and #66 KO after treatment with Brefeldin A 100 ng/mL, 5 h (BFA) or ethanol, 5 h (vehicle) under basal condition (e) or IL‐6 overexpression (h). The percentage of cell staining positive for SA‐β‐gal is shown. Bars show the mean ± SEM of three independent experiments (dots). (f, j) Cell proliferation was measured by resazurin reduction assay on IL‐6 KO clones #58 and #66 KO after treatment with BFA or vehicle under basal condition (f) or IL‐6 overexpression (j). Dots in the bar chart show MFI ± SEM of three experiments (*n* = 4) with similar results (g, i) Immunoblots of p16, pRb, pSTING, and STING of #66 KO clone extracts after treatment with BFA or vehicle under basal condition (g) or IL‐6 overexpression (i). Quantification of pSTING shows the ratio of pSTING/STING. GAPDH was used as a loading control. (k, l) Representative brightfield images of SA‐β‐gal staining, IL‐6‐GFP or IL‐6 immunofluorescence confocal images and senescence biomarkers immunoblots of IL‐6 KO clones transfected with IL‐6‐GFP‐KDEL or empty vector (clone #58) (k) or IL‐6‐KDEL, IL‐6 or empty vector (clone #66) (l). Blots are representatives of three independent experiments with similar results. GAPDH was used as a loading control. Bars show mean ± SEM quantification of independent (dots) immunoblots relative to vehicle‐treated samples (g, i, k, l). Scale bar, 100 μm (c, e, h, k, l). ***p < 0.001, **p < 0.01, *p < 0.05, ns, not significant. *p* value was calculated using an unpaired Student's *t* test or where indicated, ANOVA.

To confirm that the intracellular actions after different treatments were due to endogenous IL‐6 we generated IL‐6 deficient MtT/S cells by CRISPR/Cas9‐mediated gene deletion. We selected two full knock outs clones (#58 and #66) and one with very low IL‐6 levels (Intermediate clone), which were verified by sequencing (Figure S3) and protein expression **(**Figure 4a). We observed a significant increase in cell proliferation (Figure 4b) and the absence of SA‐β‐Gal positive staining (Figure 4c) in the IL‐6 knock out clones compared with wild type (WT) MtT/S. To evaluate the tumorigenic capacity in vivo, we injected WT and IL‐6 deficient clones into NOD/SCID mice. As previously reported, WT cells were not able to form tumors (Graciarena et al., 2004). Remarkably, both IL‐6 knock out clones developed visible tumors from day 7, while the intermediate clone formed smaller tumors 20 days after injection (Figure 4d).

Should our intracellular IL‐6‐signaling hypothesis be correct, the inhibition of the secretory pathway by BFA would not affect senescence in the IL‐6 knock out clones. We therefore set out to determine senescence biomarkers and STING signaling pathway activation in the IL‐6‐knock out. Indeed, the observed absence in SA‐β‐Gal positive cells was not modified by BFA (Figure 4e and Figure S4a). No differences were observed in proliferation, p16, pRb, and STING in the clones upon BFA exposure (Figure 4f,g and Figure S4b). When IL‐6 expression was re‐established by transfection with an IL‐6 expression vector, knock out clones showed a similar response to BFA to that observed in WT cells: SA‐β‐Gal staining and p16 expression were increased and the phosphorylation of Rb decreased (Figure 4h,i and Figure S4c,d). These results reinforce the concept that BFA does not have a general nonspecific action, but acts on senescence when IL‐6 is present. Proliferation diminished in both clones under this treatment as well (Figure 4j) and there were no differences in the ER stress marker CHOP (Figure S4e).

We next used two different plasmids coding for an IL‐6 protein that is not secreted, both carrying an endoplasmic reticulum signal—KDEL—in the C‐terminal (Rose‐John et al., 1993; Zuo et al., 2021). When IL‐6 expression was re‐established by transfection with either of the plasmids, we observed an increase in the senescence biomarkers, evidenced by the number of SA‐β‐gal positive cells and the expression levels of p16 and pRb (Figure 4k,l). The intracellular IL‐6 was observed by WB and confocal microscopy with both plasmids showing, as previously described (Rose‐John et al., 1993; Zuo et al., 2021), a diffuse IL‐6 distribution (Figure 4k,l) and was not detected in the supernatant.

Altogether, these results demonstrate that endogenous IL‐6 is crucial for the development of the senescence phenotype in MtT/S cells and that this IL‐6‐mediated senescence restricts tumorigenic potential in MtT/S cells.

### 3.5 Pro‐senescence intracellular IL‐6 signaling in glioblastoma, pulmonary, and melanoma senescent cells

To examine whether the IL‐6 intracellular signaling found in pituitary tumor cells is also occuring in other senescence models we studied Doxorubicin (Dox)‐treated lung adenocarcinoma A549 (Yun et al., 2012) and glioblastoma U87MG (Gu et al., 2021) cells and Cisplatin‐treated melanoma A375 (Kuilman et al., 2008), as models of therapy‐induced senescence in which IL‐6 has been demostrated to be expressed and regulates proliferation. In Dox‐treated A549 cells, IL‐1β was used to boost IL‐6 production (Eda et al., 2011) and thus the intracellular accumulation after BFA treatment.

**FIGURE 5:**
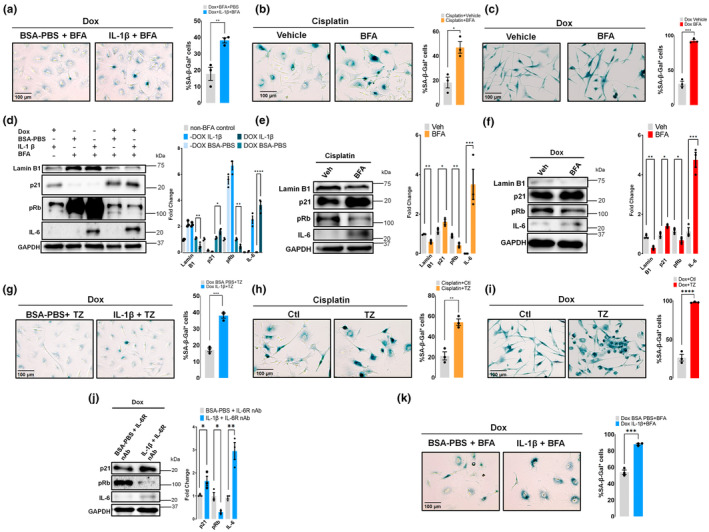
Senescent effect of intracrine IL‐6 in three cellular models of therapy‐induced senescence. (a–c) Representative brightfield images of SA‐β‐gal staining of lung adenocarcinoma senescent A549 cells treated with 132 nM doxorubicin (Dox) for 48 h, 20 ng/mL IL‐1β or BSA‐PBS for 24 h and with Brefeldin A 100 ng/mL, 5 h (BFA) (a), senescent melanoma A375 cells treated with 2 μM Cisplatin for 24 h and cultured 7 days with a drug‐free medium and with BFA or vehicle (ethanol, 5 h) (b) or senescent glioblastoma U87MG cells treated with 100 nM dox for 48 h and with BFA or vehicle (c). The percentage of cell staining positive for SA‐β‐gal is shown. Bars show the mean ± SEM of three independent experiments (dots). (d–f) Immunoblots of lamin B1, p21, pRb, and IL‐6 of proliferating and senescent A549 cells (d) treated as described in “a”, senescent A375 cells (e) treated as described in “b” and senescent U87MG cells (f) treated as described in “c” (g–i) Representative brightfield images of SA‐β‐gal staining of senescent A549 cells treated as described in “a” after 48 h treatment with 100 ng/mL tocilizumab (TZ) (g), senescent A375 cells treated as described in “b” after 72 h treatment with 100 μg/mL TZ (h) and senescent U87MG treated as described in “c” after 48 h treatment with 10 μg/mL TZ or isotype control (Ctl). (i) The percentage of cell staining positive for SA‐β‐gal is shown. Bars show the mean ± SEM of three independent experiments (dots). (j) Immunoblots of p21, pRb, and IL‐6 of senescent A549 treated as described in “a” in which human IL‐6 receptor neutralizing antibody was added to the cell culture media (10 μg/mL, 48 h). Blots are representatives of three experiments with similar results. GAPDH was used as a loading control. (k) Representative brightfield images of SA‐β‐gal staining of senescent A549 cells treated with 132 nM Dox for 48 h and cultured 7 days with a drug‐free medium, 20 ng/mL IL‐1β or BSA‐PBS for 24 h and with BFA. The percentage of cell staining positive for SA‐β‐gal is shown. Bars show the mean ± SEM of three independent experiments (dots). Scale bar, 100 μm (a–c, g–i, k). ****p < 0.0001, ***p < 0.001, **p < 0.01, *p < 0.05, ns, not significant. *p* value was calculated using an unpaired Student's *t* test or where indicated, ANOVA.

We observed an increase in the number of SA‐β‐Gal‐positive cells in senescent A549 and U87MG cells after 48 h Dox treatment and in A375 cells exposed to Cisplatin for 24 h and analyzed 7 days after exposure (Figure 5a–c), when intracellular IL‐6 was increased by BFA. Senescence biomarkers p21, pRb, and lamin B1 changed according to the increase in senescence and IL‐6, detected by WB in all senescent cell types (Figure 5d–f). In A549 cells treated with IL‐1β and BFA, IL‐6 induced the senescence biomarkers only in Dox‐treated cells, indicating that senescence may be a requirement to observe the differential intracellular effects of this signaling pathway (Figure 5d). As expected, Dox reduced the proliferation of U87MG cells and the treatment with BFA reduced it even further (Figure S5a).

After the neutralization of membrane IL‐6R with tocilizumab (TZ), which we checked that blocks exogenous IL‐6‐induced pSTAT3 phosphorylation levels (Figure S5b–d), we still observed the increase in the number of SA‐β‐Gal positive cells in the three senescent cell lines (Figure 5g–i). We also used another neutralizing antibody against human IL‐6R—which blocks exogenous IL‐6‐induced STAT3 phosphorylation levels (Figure S5e)—in cells in which endogenous IL‐6 production was stimulated with IL‐1β, that resulted, without the addition of nonspecific blockers, in an induction of senescence biomarkers similar to the one obtained with the intracellular accumulation of IL‐6 caused with BFA treatment (Figure 5j). These experiments confirm that the senescent action does not involve the extracellular signaling through IL‐6R.

In A549 cells cultured also for 7 days after Dox treatment, we observed a similar pattern for SA‐β‐Gal staining, confirming the role of intracellular IL‐6 in stably senescent cells (Figure 5k).

**FIGURE 6:**
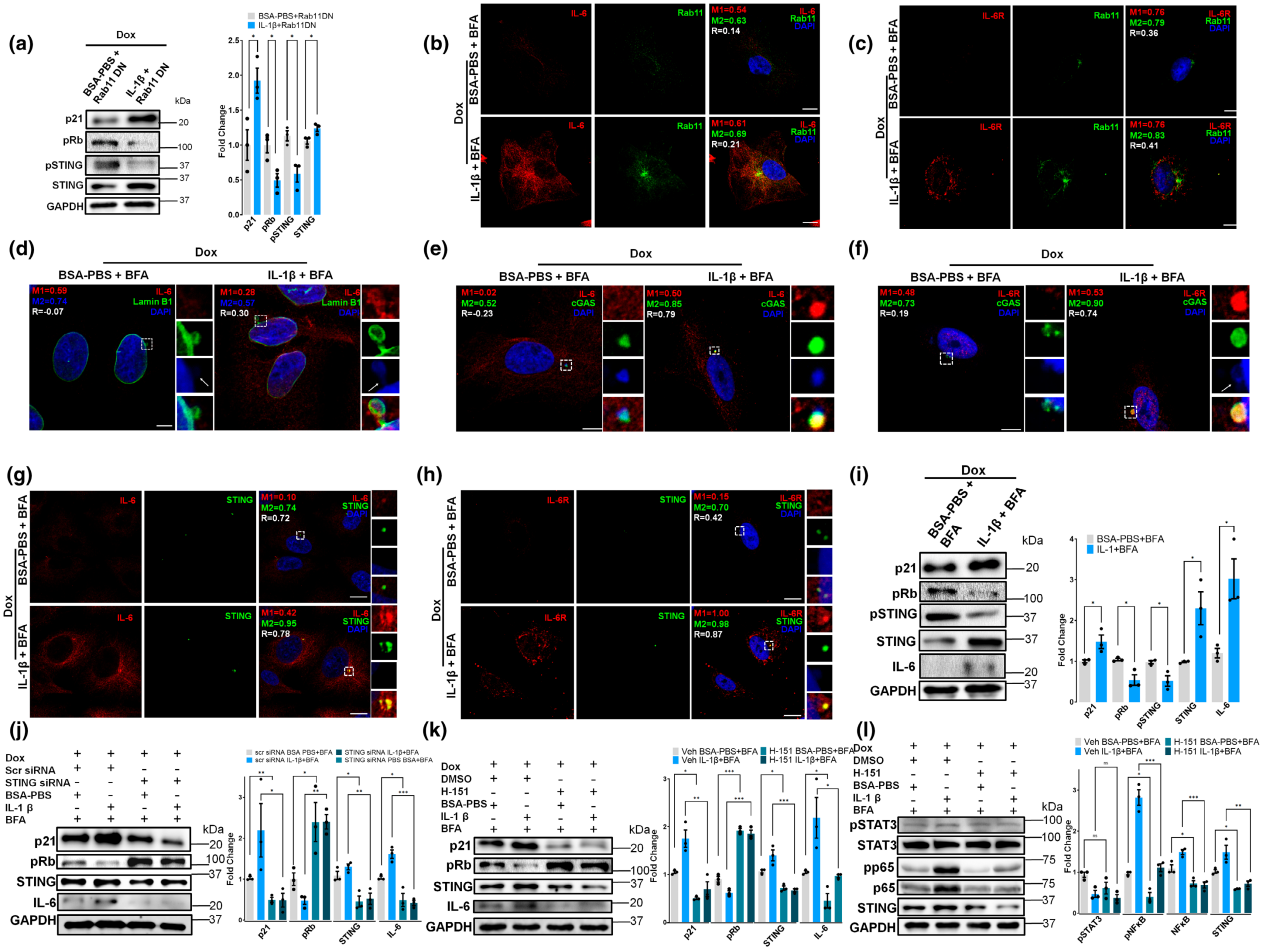
Intracellular action of IL‐6 in doxorubicin‐induced senescent A549 cells. (a, i) Immunoblots of p21, pRb, IL‐6, pSTING, STING of senescent A549 cells treated with Brefeldin A 100 ng/mL, 5 h (BFA) or 20 ng/mL IL‐1β or BSA‐PBS for 24 h. GAPDH was used as a loading control. To induce senescence 132 nM doxorubicin (Dox) was used for 48 h. In “a” A549 cells were transfected with a plasmid coding for a dominant negative form of Rab11 before Dox was added. (b–h) Representative immunofluorescent confocal images using specific antibodies against Rab11, Lamin B1, cGAS, or STING of senescent A549 cells treated with 132 nM Dox for 48 h, BFA or 20 ng/mL IL‐1β or BSA‐PBS for 24 h. DAPI (blue) was used to stain cell nuclei. DAPI‐stained fragments are indicated by white arrows. Manders coefficients for each channel (M1 and M2) and Pearson's correlation coefficient (R) were calculated in cell cytoplasm (b, c), excluding the nuclei, for each condition (8–12 cells per experiment) and in zoomed views of selected regions of interest (d–h). Images are representatives of three independent experiments with similar results. Scale bars = 10 μm (j–l) Immunoblots of p21, pRb, IL‐6, STING, pSTAT3, STAT3, p65, and pp65 of senescent A549 cells extracts treated with scramble or STING siRNA (j), STING antagonist H‐151 (5 μM) or vehicle (DMSO) for 5 h in combination with BFA (k, l). Quantification of pSTAT3/STAT3 ratio is shown in “l”. GAPDH was used as a loading control. *p* value was calculated using ANOVA, followed by Tukey test. Blots are representatives of three experiments with similar results. GAPDH was used as a loading control. Bars show mean ± SEM quantification of independent (dots) immunoblots relative to vehicle‐treated samples (a, i–l). ***p < 0.001, **p < 0.01, *p < 0.05, ns, not significant. *p*‐value was calculated using an unpaired Student's *t* test or where indicated, ANOVA.

We further studied in detail the IL‐6 intracellular signaling pathway in A549 cells, in which IL‐6 has a dual role in tumorigeneis (Yun et al., 2012). As expected, overexpression of dominant negative form of Rab11, which promotes IL‐6 accumulation (Figure S5f) had similar effect on senescence biomarkers as BFA (Figure 6a). Similar to what we observed in MtT/S cells, IL‐6 and IL‐6R are spatially associated with Rab11 and this association persisted with the increase in IL‐6 production in A549 senescent cells (Figure 6b,c). Altogether, these results indicate that the intracrine action of IL‐6 is critical for the modulation of senescence biomarkers in a therapy‐induced senescent model.

We next explored lamin B1 structure and cGAS distribution connected to the presence of chromatin fragments. When IL‐6 was induced, nuclear membrane blebs were detected linked to IL‐6 signal **(**Figure 6d) and we noted intense punctuated dots, in the areas where IL‐6 and IL6R co‐localized with cGAS and STING (Figure 6e–h).

Intracellular IL‐6 accumulation induced by BFA or the overexpression of the Rab11 dominant negative was accompanied by increased total and decreased phosphorylated STING levels (Figure 6a,i). STING knockdown or inhibition with H‐151 abolished the senescent phenotype (Figure 6j,k). STING inhibition also abolished NFκB activation, but had no effect on pSTAT3 phosphorylation (Figure 6l) confirming that STAT3‐dependent signaling is not involved in intracellular senescent signaling.

Altogether our results demonstrate that in therapy‐induced senescence as well as in naturally senescent tumor cells IL‐6 modulates senescence via intracrine signaling that involves cGAS‐STING and the NFκB pathway.

## 4 DISCUSSION

SASP factors, particularly IL‐6, are pleiotropic and can impact on various biological processes. They can transmit cellular senescence to normal neighboring cells in a paracrine manner (Acosta et al., 2013). Paradoxically, the SASP has also pro‐tumorigenic properties (Coppe et al., 2010), as they can be involved in paracrine tumor promotion. The pro‐ tumorigenic and antitumorigenic properties of the SASP confer diversity to the senescent phenotype and its functional output beyond growth arrest. The intracellular mechanism described in this paper adds a new level of complexity to the action of the SASP factors.

The opposing role of IL‐6 in cellular senescence and tumorigenesis is well represented in pituitary tumors development. Pituitary tumorigenesis appears as a complex process in which extrinsic and intrinsic factors contribute to sustain cell proliferation (Melmed, 2011). Pituitary tumors present a natural model in which senescence controls its expansion and explains why these tumors have a benign nature (Chesnokova et al., 2007). In these processes IL‐6 acts as a double‐function key protein in the control of senescence and tumorigenesis.

IL‐6/STAT3 canonical signaling triggered by IL‐6 membrane receptor interaction has proliferative effects on human pituitary adenomas, pituitary stem cells, and pituitary tumor cell lines together with tumor associated‐fibroblasts (TAF) in the microenvironment of pituitary tumors (Graciarena et al., 2004; Marques et al., 2019; Sapochnik et al., 2017). Accordingly, infrequent malignant‐related features of these tumors have been reported with increased IL‐6 secretion levels and STAT3 activation (Cai et al., 2019).

Cytokines may act on the cells that produce them in an autocrine manner upon binding to their cell surface receptors. IL‐2 has been found to act through membrane receptor‐independent signaling (Volko et al., 2019). Recent work on dendritic cells showed that exogenous IL‐6 is internalized by these cells and signals from endosomal compartments that contain its receptor (Verboogen et al., 2018). It is clearly established that the endosomal system is involved in generating and modulating signaling (Acosta et al., 2013). The experiments blocking endocytosis show that this is not the case for the action of IL‐6 in senescence. In the dendritic cell model, it has been shown that newly synthesized IL‐6 that travels through intracellular structures also activates STAT3 in transit to the plasma membrane (Verboogen et al., 2018). In human mesenchymal stromall cells (hMSC) increasing the intracellular level of IL‐6 could restore the proliferative impairment observed in IL‐6‐silenced hMSC (Dorronsoro et al., 2020). These findings are in line with the notion that the same protein can activate signaling cascades from different regions of the cell (Drenan et al., 2004).

The endogenous intracrine action of senescence‐inducing IL‐6 was initially observed by inhibiting secretion and also endocytosis. Inhibition of secretion was performed pharmacologically by BFA as well as by means of Rab11 molecular blockage. Rab11 is described as a key component of exocytic pathway and is associated with anterograde trafficking (Sorvina et al., 2020). Intracellular retention of IL‐6 (specifically confirmed in IL‐6 knock out cells with an expression vector coding for an IL‐6 protein that is not secreted and thus without it being able to bind to the receptor expressed in the membrane) and blocking the endocytosis of the IL6/IL‐6R/gp130 complex—necessary for triggering proliferative signals—induced senescence markers expression and decreased STAT3 phosphorylation. Neutralization of human cell membrane IL‐6R in therapy‐induced senescent cells indicates that the senescent action that we observe when IL‐6 accumulates is of intracellular, not external by means of autocrine or paracrine origin, independent of the secretion and of the participation of the membrane receptor. Alltogether these results using different experimental tools, indicate that the endogenous action of IL‐6 not only uses an alternative route to the canonical one but also that its intracellular location is necessary. The inhibition of endocytosis indicate that the IL‐6/IL‐6R does not use the pathways coupled to endocytosis, but rather those associated with the secretory pathway. Similarly to the endosomal system, the organelles involved in the secretory pathway contribute not only to signaling but also to the compartmentalization of signal transduction to trigger different cellular responses, such as cell proliferation, transformation, and migration (Choudhary et al., 2009; Obata et al., 2014) and, as we demonstrated for the first time in this work, cellular senescence signals.

During the process of cellular senescence extracellular vesicles (EV) are actively generated (Wallis et al., 2020). It was reported that the decrease in the elimination of exosomes that contain DNA enhance senescence in both senescent and non‐senescent models (Takahashi et al., 2017) supporting our findings in which the inhibition of the secretion increases senescence in different senescent tumoral models. Moreover, pharmacological or molecular inhibition of exosome secretion by downregulating Rab27a, induced the same effects (Takahashi et al., 2017), indicating that these senescent actions are not dependent on the molecular mechanism that restrict secretion as we observed with BFA and overexpressing a dominant negative form of Rab11.

We observed that IL‐6, together with its receptor are spatially organized in subcellular locations linked to anterograde traffic, from where they might induce pro‐senescent signals. The colocalization in intense punctuated dots was clearly evidenced for endogenous proteins in A549 cells. We noted a senescent phenotype mediated by the cGAS‐STING pathway when IL‐6 levels accumulate intracellularly, avoiding the interference of IL‐6‐canonical STAT3 participation, in both senescent A549 cells and pituitary MtT/S, evidencing cGAS‐STING pathway contribution in pituitary tumors.

The activation of STING is triggered in most of the cases by the DNA sensor cGAS both in inflammatory and noninflammatory conditions (Li & Chen, 2018). Within the senescent phenotype DNA fragments leak through the nuclear lamina and are found in the cytoplasm named cytosolic chromatin fragments (CCF) (Dou et al., 2017). This phenomenon can be explained by the loss of nuclear envelope integrity product of lamin B1 degradation in specific areas and observed by the presence of nuclear blebbing. In MtT/S and senescent A549 cells we noted images compatible with CCF as we detected DAPI‐stained fragments in confocal images and nuclear envelope blebbing by immunofluorescence of lamin B1 together with cytosolic chromatin protrusion. Accumulation of CCF had not been detected in pituitary tumors before. We uncovered in this work the intersecting signals of IL‐6/IL‐6R with cGAS‐STING and CCF. The adjacent structures throughout the secretory pathway may represent a specific subcellular place in which the intracrine senescent signal is triggered from. Considering that BFA blocks ER‐Golgi communication and a recent work that described NFkB activation by STING previous of its traffic to the Golgi (Stempel et al., 2019), STING could signal independently of the type I IFN response associated to the intracellular IL‐6 from specific location in close proximity to the ER, as very recently described in a type I interferonopathy (Hirschenberger et al., 2023). Since nuclear blebbing is a common feature of cellular senescence that we observed in the conditions where intracellular IL‐6 accumulates or not, we hypothesize that once a tumoral cell enters the senescence state either by OIS or TIS, intracellular IL‐6 could act as an amplifier of the senescent signal coupling IL‐6R to CCF and cGAS‐STING pathway with the downstream activators involved in the maintenance of the SASP. The term *IL‐6 amplifier* is not a novel concept as it has been used to describe the positive feedback loop of IL‐6 activation by NFkB and STAT3 in immune and nonimmune cells (Hirano, 2021).

Senescent cells express SASP components that promote SASP‐mediated signaling events by binding to their respective receptors. By this way, senescent cells communicate among themselves or induce senescence on other target cells that express its receptor via paracrine activity, the expression levels of the SASP component receptors on the target cell being one of the factors determining the outcome of the response. The observed action of the SASP component signaling on the senescent cell itself via autocrine activity, lead to the question of the mechanism of action, which we describe for IL‐6, one of the key SASP members. Interestingly, the initial trigger for the IL‐6R activation can originate inside the IL‐6 producing cell (cytosolic DNA). In senescent cells, E2F transcription factor is suppressed which results in the suppression of TREX1 (DNAse3) responsible for degradation of self‐DNA cytoplasmic fragments which accumulate triggering activation of cGAS‐STING pathway to promote SASP cytokines including IL‐6 (Takahashi et al., 2018). Downstream of cGAS and STING, similar to other senescent pathways, the signals converge to disable the NF‐κB inhibitor IκBα, activating this master transcription factor in the senescence response (Lopes‐Paciencia et al., 2019).

Senolytic therapy has been a promising treatment against cancer development and spread (Wang et al., 2022). Special care should be taken with senotherapeutic drugs, especially with senomorphics that may act on IL‐6 signaling considering that pituitary tumors are very frequent and the induction of the senescence program strongly dependent on IL‐6 signaling represents a protective mechanism against oncogenic activation. The acquisition of tumorigenic capacity in vivo of IL‐6 knock out MtT/S cells clearly shows the relevance of this cytokine in pituitary tumor senescence. The use of extracellular vesicles as highly effective senotherapeutic (Dorronsoro et al., 2021) may be a very interesting approach for IL‐6 based senolytic therapy. Pharmacological inhibition of coatomer protein complex I (COPI) results in Golgi dispersal, dysfunctional autophagy, and unfolded protein response (UPR)‐dependent apoptosis of senescent cells. Drugs targeting the COPI have poor pharmacological properties, but it was very recently described that *N*‐myristoyltransferase inhibitors (NMTi) phenocopy COPI inhibition and are potent senolytics, acting through the inhibition of GBF1, a guanine nucleotide exchange factor required to activate ARF essential for the formation of COPI vesicles (McHugh et al., 2023). Cells undergoing OIS showed an increase in the intracellular levels of cytokines such as IL‐8 or IL‐6, and the levels of IL‐8 and IL‐6 were much higher in senescent cells upon *COPB2* depletion, the treatment with GBF1 inhibitors causing the intracellular accumulation of other SASP components such as VEGF, GM‐CSF, and BMP2/4 in senescent cells (McHugh et al., 2023). The senolytic effects of GBF1 inhibitors are reversible and prevented if the drugs are removed 24 h, but not 48 h, post treatment, indicating that the initial steps of protein accumulation are not critical for the action. Interestingly, aberrant accumulation of the SASP (and other misfolded proteins) on senescent cells could trigger a UPR, which may contribute to the senolytic effects associated with *COPB2* knockdown (McHugh et al., 2023). Adjusted pharmacological disruption of the cellular senescent pathways, including the SASP paracrine and intracellular IL‐6 pathway's action, are critical for the effective senolytic action. The mechanism we describe might provide therapeutic options in which membrane‐receptor driven IL‐6 signaling is blocked, preventing growth stimulation, but intracellular IL‐6 signaling remains intact, enhancing senescence‐induction.

In this work, we describe an intracellular senescent signaling of IL‐6 in a natural and three therapy‐induced senescent models and we characterize the molecular intermediates that trigger and amplify this signal. IL‐6 is induced in the tumor cells and, together with its receptor—with which the spacial coupling occurs in some places of the intracellular compartments linked to the anterograde transport during its traffic to the membrane—amplifies the activation of a senescent response carried out via cytosolic DNA and cGAS‐STING pathway. This intracrine signaling cascade increases the expression of NFκB, essential regulator of the SASP and in particular of IL‐6, which reinforces the pro‐senescent signals and drives the SASP, allowing this phenotype to be maintained over time. Since the pro‐tumor and pro‐senescent action of IL‐6 (and other SASP factors) is widely described in multiple tumors, the new mechanism of action of IL‐6 that contributes to discriminate these signals discovered in these tumor models could very likely represent a general mechanism for other senescent cells.

## AUTHOR CONTRIBUTIONS

E.A was involved in conceptualization; F.H, M.S, B.E, M.F, L.B.P, and E.A were involved in project administration; E.A was involved in supervision; F.H, M.S, A.A, C.P, S.S, D.G.P, N.C.G, M. Fiz, L.M, and M.T were involved in investigation. F.H, M.S, B.E, M.F, M.T, L.B.P, and E.A were involved in formal analysis; E.A. was involved in funding acquisition; F.H, M.S, and E.A were involved in writing original‐draft; L.B.P and E.A. were involved in writing‐review and editing. All the authors read and approved the final manuscript.

## FUNDING INFORMATION

This work was supported by grants from the Max Planck Society, Germany (2012); the University of Buenos Aires (grant number 20020170100230BA); CONICET (grant number PUE‐2016 (22920160100010CO)); the Agencia Nacional de Promoción Científica y Tecnológica (ANPCyT) (grants number PICT2016‐1620 and PICT‐2018‐03232), Argentina; and FOCEM‐Mercosur (COF 03/11).

## CONFLICT OF INTEREST STATEMENT

The authors declare no competing interests.

## Supporting information


Appendix S1.

## Data Availability

Data available on request from the authors.
