# Supplementary material for: The SASP factor IL‐6 sustains cell‐autonomous senescent cells via a cGAS‐STING‐NFκB intracrine senescent noncanonical pathway
Source: Aging Cell. 2024 Jul 16;23(10):e14258. doi: 10.1111/acel.14258 (PMC11464112; doi:10.1111/acel.14258)

**
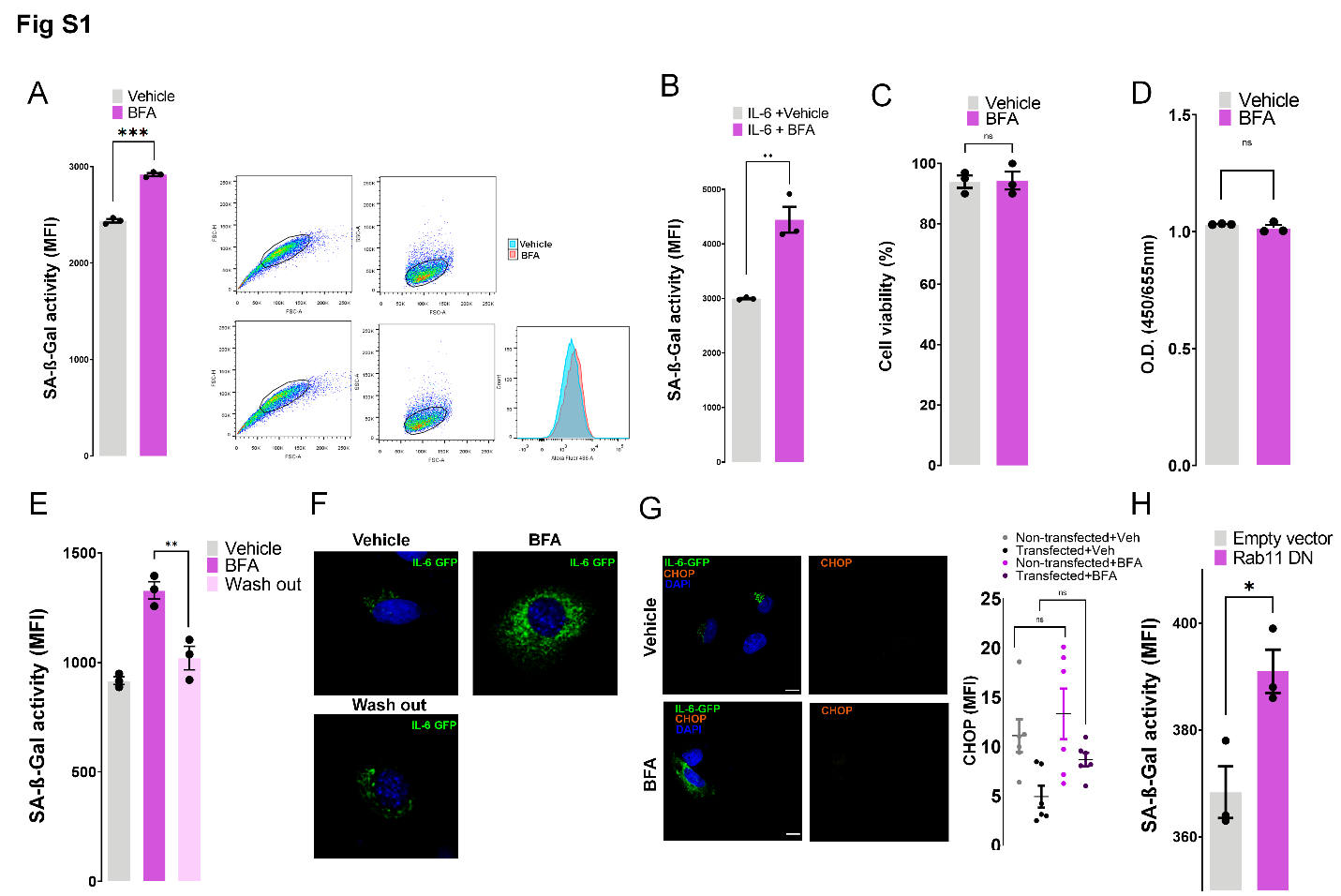
**

**FigS1 - Determination of toxicity, dose and effect of BFA in MtT/S cells**

(**A,B,H**) Senescence associated-β-galactosidase (SA-β-gal) was quantified by flow cytometry with C_12_FDG substrate. Graphs represent Mean fluorescence intensity (MFI) after treatment with Brefeldin A 100 ng/ml, 5h (BFA) or ethanol, 5h (vehicle) under basal condition (**A**), IL-6-overexpression (**B**) or after expression of a dominant negative form of Rab11 or empty vector (**H**). In **A** Flow cytometry FSC-A vs FSC-H, FSC-A vs SSC-A dot plots and overlay histogram for SA-β-Gal fluorescent staining for vehicle and BFA treatments are shown. Bars show the mean ± SEM of three independent experiments (dots). (**C-D**) Cell viability by Trypan Blue dye staining (**C**) and cell proliferation by WST-1 assay (**D**) were measured to verify that the used dose and time of BFA had no toxicity. MtT/S cells were previously treated with BFA or vehicle. (**E-F**) SA-β-gal was quantified by flow cytometry with C_12_FDG substrate (MFI) (**E**) and confocal microscopy of cells transfected with IL-6-GFP (green) (**F**) were measured to check for the reversible effect on BFA on the disruption of the Golgi apparatus. MtT/S cells were treated with BFA or vehicle and BFA was washed out for another 5h. Bars show the mean ± SEM of three independent experiments (dots). (**G**) Representative immunofluorescent confocal images using specific antibodies against CHOP of MtT/S cells transfected with expression vector of IL-6 fused to GFP (IL-6-GFP) (green) and treated with BFA or vehicle. DAPI (blue) was used to stain cell nuclei. MFI of CHOP is shown for each condition. ***p <0.001, **p<0.01, *p<0.05, ns, not significant. p-value were calculated using an unpaired Student’s t-test. Scale bars = 10μm (**F,G).**


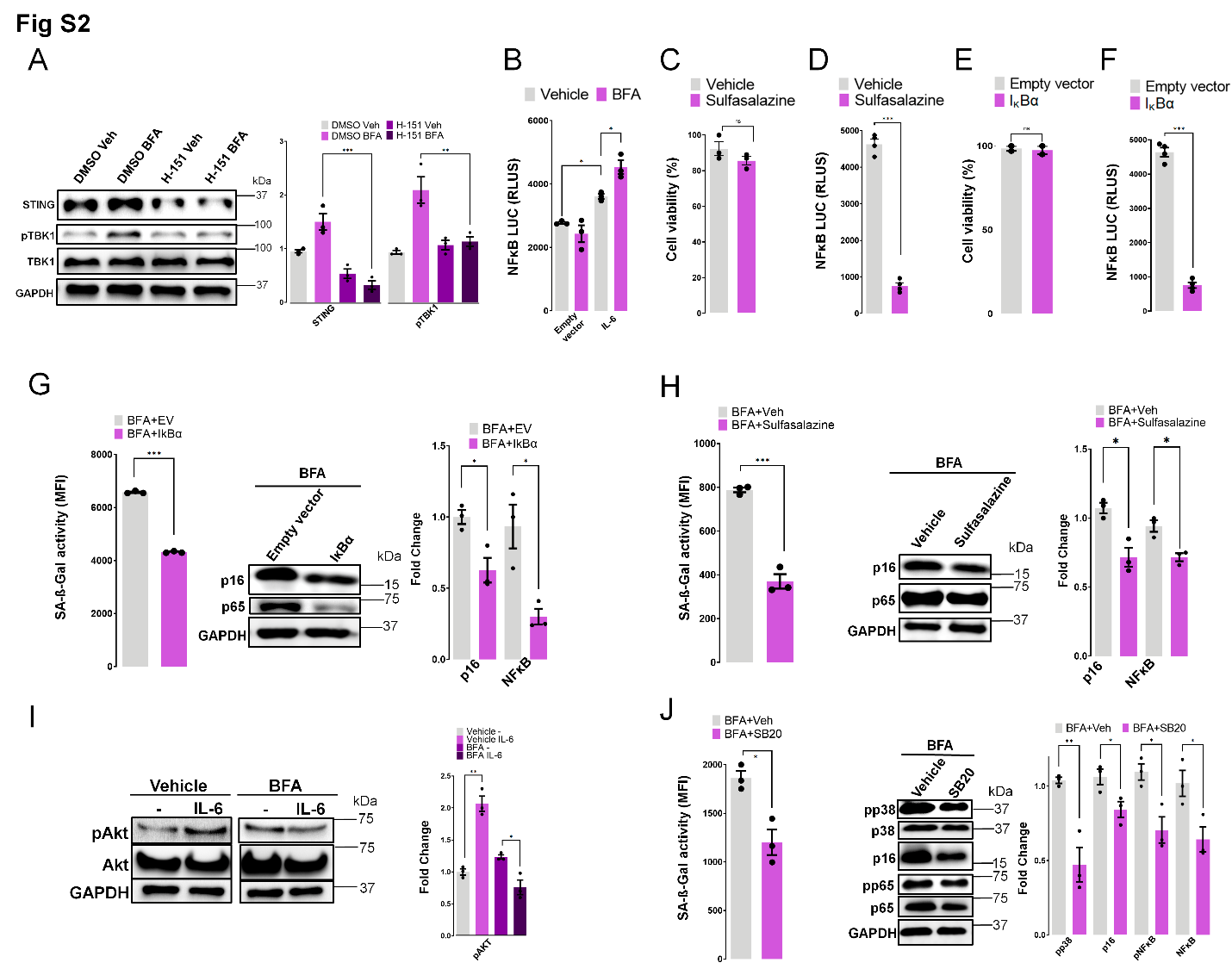


**FigS2 - The intracellular senescent IL-6 signaling is coupled to NFκB and p38MAPK signaling**

(**A**) Immunoblots of STING, pTBK1 and TBK1 of MtT/S cells extracts treated with STING antagonist H-151 (5μM) or DMSO for 5 h in combination with Brefeldin A 100 ng/ml, 5h (BFA) or ethanol. Quantification of pTBK1/TBK1 ratio is shown. GAPDH was used as a loading control. p-value was calculated using ANOVA, followed by Tukey test. (**B**) MtT/S cells were transfected with the following vectors: 0.4μg of the activity reporter of NFκB (NFκB-LUC), 0.4 μg of IL-6-GFP or its control vector, and 0.2 μg of the reporter CMV-β-galactosidase. 48h post-transfection cells were treated BFA or vehicle and luciferase (RLU) was measured. Each value was normalized to CMV-β-galactosidase activity. Data in the bar chart show mean ± SEM of triplicates of one of two experiments (n=2) with similar results. p-value was calculated using ANOVA, followed by Tukey test. (**C-F**) Genetic (IκBα) and pharmacological (sulfasalazine) inhibition of NFκB were tested. It was verified that the dose used of sulfasalazine vs drug-free medium as vehicle (**C**) or IκBα transfection vs empty vector as vehicle (**E**) did not affect cell viability (Trypan blue dye staining) and that the functional activity of NFκB decreased by the action of these inhibitors (**D,F**). Data in the bar chart show mean ± SEM of triplicates of one of two experiments (n=2) with similar results. p-value was calculated using an unpaired Student’s t-test. (**G,H**) SA-β-gal activity was quantified in MtT/S cells transfected with 0.8μg of IκB expression vector (**G left**) or treated with sulfasalazine (3mM) for 5h (**H left**) and treated with BFA, by flow cytometry with C_12_FDG substrate. Graph represents Mean fluorescence intensity (MFI). Bars show the mean ± SEM of three independent experiments (dots). Immunoblots of p16 and pRb of extracts from MtT/S cells transfected with 0.8μg of IκB expression vector (**G right**) or treated with sulfasalazine (3mM) for 5h (**H right**) and then treated with BFA. GAPDH was used as a loading control (**I**) Immunoblots of p-Akt and Akt of extracts from MtT/S cells treated with BFA or vehicle. Quantification of pAKT/AKT ratio is shown. (**J left**) SA-β-gal activity was quantified in MtT/S cells treated with the p38MAPK inhibitor SB203580 (SB20) (5μM) or DMSO for 5h and treated with BFA, by flow cytometry with C_12_FDG substrate. Graph represents MFI. Blots are representatives of three independent experiments with similar results. GAPDH was used as a loading control. Bars show mean ± SEM quantification of independent (dots) immunoblots relative to vehicle-treated samples. p-value was calculated using an unpaired Student’s t-test. (**J right**) Immunoblots of pp38, p38, p16, pp65 and p65 of extracts from MtT/S cells treated with 5μM SB20 or vehicle for 5h and treated with BFA. Quantification of pp38 shows the ratio of pp38/p38 and for pp65 the ratio of pp65/p65. GAPDH was used as a loading control. Bars show mean ± SEM quantification of independent (dots) immunoblots relative to vehicle-treated samples.

***p <0.001, **p<0.01 *p<0.05, ns, not significant


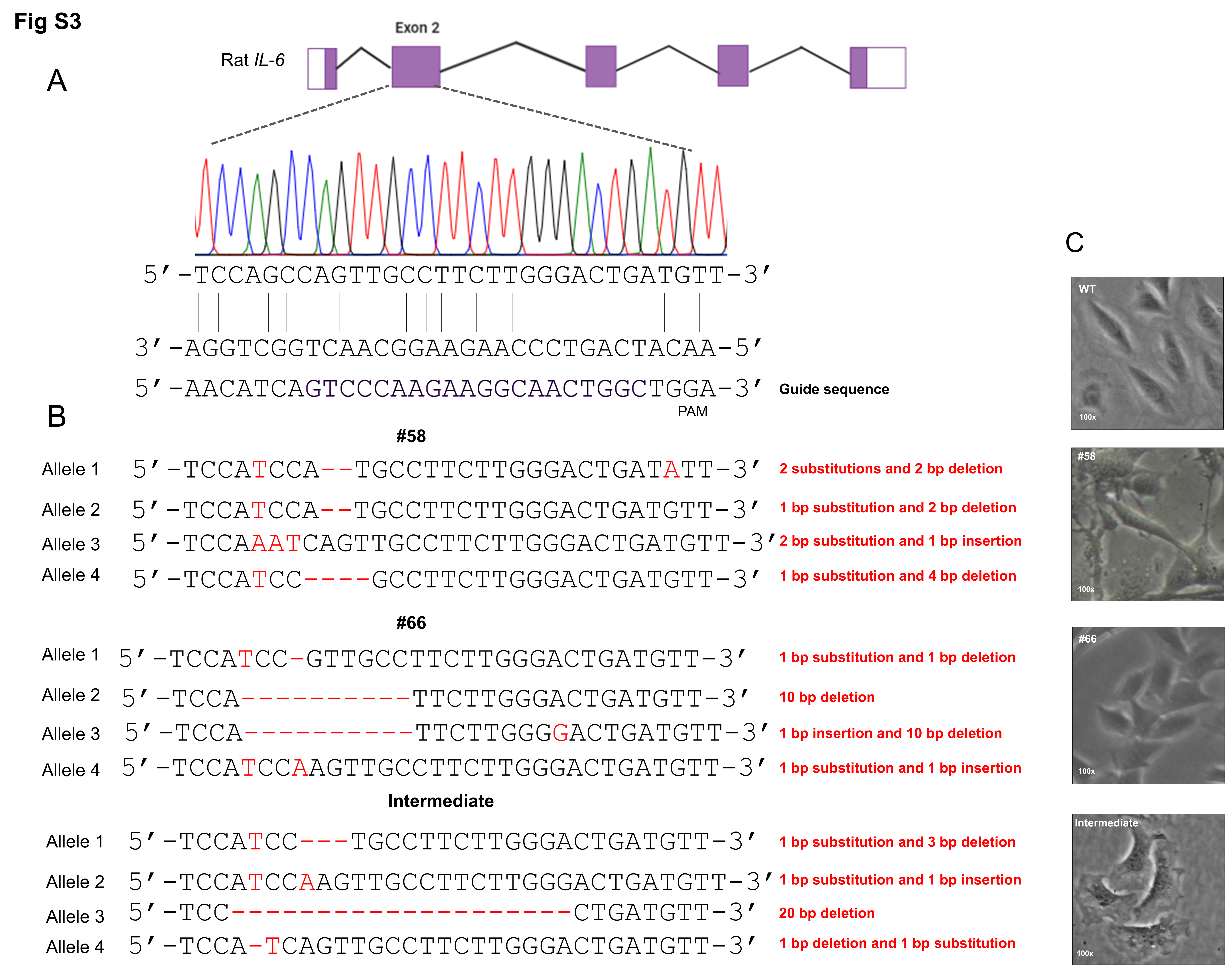


**FigS3- Generation and Validation of MtT/S IL-6 knockout cell lines**

(**A**) Top: schematic representation of the target sequence in exon 2 of Rat IL-6 gene. Bottom: sequence of the sgRNA target site is lettered in purple and the PAM motif is underlined. (**B**) CRISPR/Cas9 gene editing mutations at exon 2 in the IL-6 gene of the clones used in this work: Four different types of mutations were found, which are described to the right of the sequences. Deletions are shown with hyphens and insertions or substitutions nucleotides are in red. Considering that MtT/S cell line was reported to contain 42 chromosomes, independently of the number of IL-6 gene copies or the clonality of the generated cell lines, both clones 58 and 66 show no expression of IL-6 (**Fig 4A**). We have included a clone called “intermediate” that also shows modifications in the 4 alleles, tough one of them is a 3bp (in-frame) deletion. Accordingly, this “intermediate” clone shows partial expression of IL-6, and indeed it shows intermediate phenotypes (**Fig4 A-C**). (**C**) Representative photographs of wild type (WT) MtT/S and IL-6 deficient cell clones obtained by CRISPR/Cas9. Original magnification is 100x.


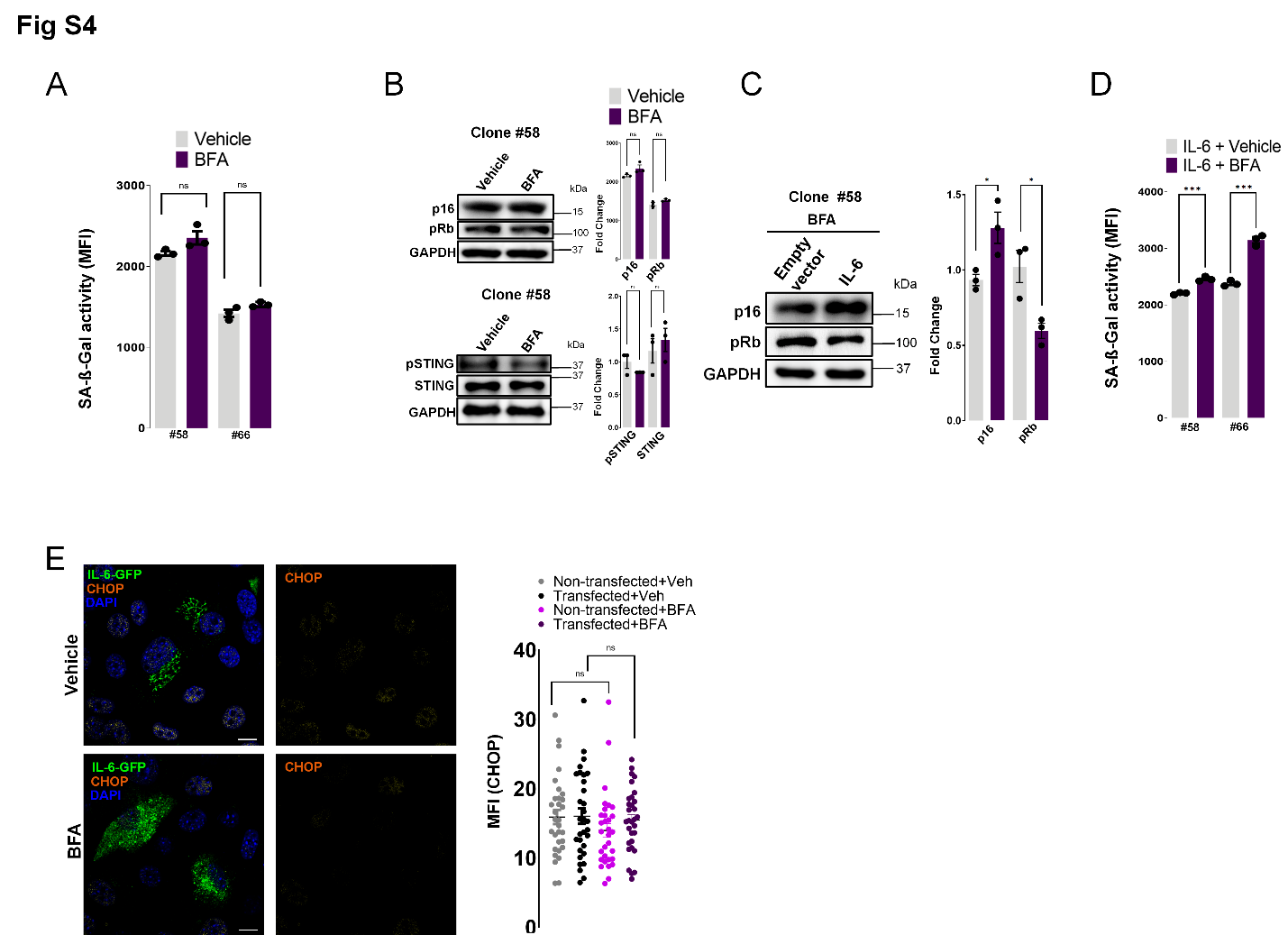


**FigS4-Intracellular senescent action of IL-6 in IL-6 KO clones**

(**A,D**) SA-β-gal was quantified by flow cytometry with C_12_FDG substrate. Graph represents Mean Fluorescence Intensity (MFI) after Brefeldin A 100 ng/ml, 5h (BFA) or ethanol, 5h (vehicle) of #58 and #66 KO clones under basal condition (**A**) or IL-6 overexpression (**D**). Bars show the mean ± SEM of three independent experiments (dots). (**B,C**) Immunoblots of p16, pRb, pSTING and STING of extracts from #58 KO clone cells under basal conditions (**B**) or transfected with IL-6 or with empty vector (**C**) treated with BFA or vehicle. Quantification of pSTING shows the ratio of pSTING/STING. (**E**) Representative immunofluorescent confocal images using specific antibodies against CHOP of #66 IL-6 KO clone MtT/S cells transfected with expression vector of IL-6 fused to GFP (IL-6-GFP) (green) and treated with BFA or vehicle. DAPI (blue) was used to stain cell nuclei. Mean fluorescence intensity (MFI) of CHOP is shown for each condition.

Blots are representatives of three experiments with similar results. GAPDH was used as a loading control. Bars show mean ± SEM quantification of independent (dots) immunoblots relative to vehicle-treated samples (**B,C**).

***p<0.001, *p<0.05, ns, not significant. p-value was calculated using an unpaired Student’s t-test or where indicated, ANOVA.

**
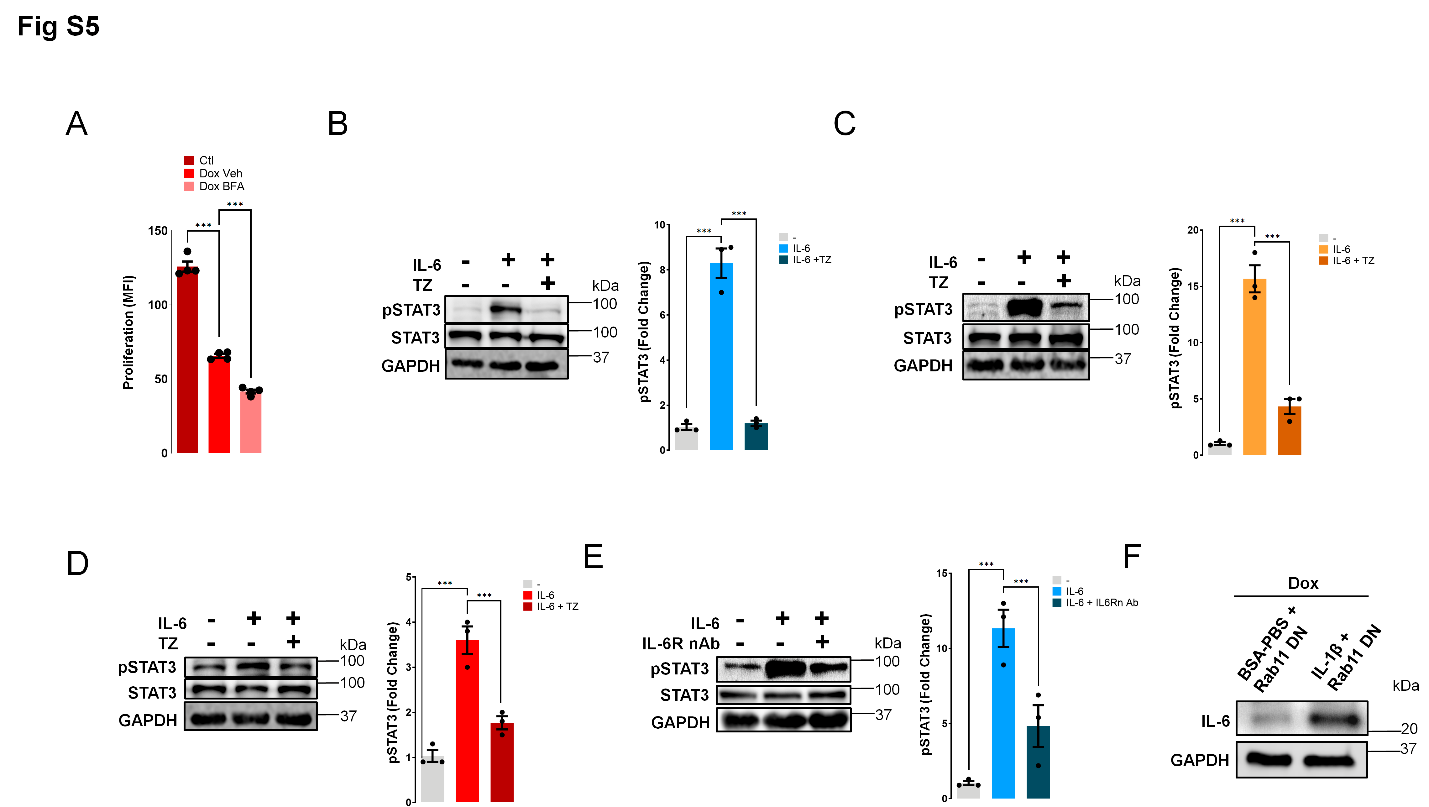
**

**FigS5- Proliferation, human IL-6 membrane receptor antibody and IL-6 expression in human senescent cells**

(**A**) Cell proliferation was measured by resazurin reduction assay on proliferating (ctl) or senescent U87MG cells. Senescence was induced by the treatment with 100 nM doxorubicin (Dox) for 48 hours and cells were exposed to Brefeldin A 100 ng/ml, 5h (BFA) or ethanol, 5h (vehicle). Bars show Mean Fluorescence Intensity (MFI) ± SEM of quadruplicates of one of three experiments (n=3) with similar results. (**B-D**) Immunoblot of pSTAT3 and STAT3 of A549 (**B**), A375 (**C**) or U87MG (**D**) cells extracts treated with tocilizumab (TZ) which was added to the cell culture medium for 48 h at a concentration of 10 ng/ml (**B**), 72 h at a concentration of 100 μg/ml (**C**) or 48 h at a concentration of 10 μg/ml (**D**) to test the capacity to neutralize the phosphorylation of STAT3 as consequence of IL-6 interaction. Recombinant human IL-6 was used (50 ng/ml) for 30 min. (**E**) Immunoblot of pSTAT3 and STAT3 of A549 cell extracts treated by human IL-6 receptor neutralizing antibody (IL-6Rn Ab) which was added to the cell culture medium for 48 hours at a concentration of 10 μg/ml to test the capacity to neutralize the phosphorylation of STAT3 as consequence of IL-6 interaction. Recombinant human IL-6 was used (50 ng/ml) for 30 min. Quantification of immunoblots are shown relative to vehicle-treated samples. p-value was calculated using ANOVA, followed by Tukey test. Quantification of pSTAT3 shows the ratio of pSTAT3/STAT3 (**B-E**). (**F**) Immunoblot of IL-6 of A549 cells transfected with a plasmid coding for a dominant negative form of Rab11 before Dox 132 nM was added to induce senescence. Bars show mean ± SEM quantification of independent (dots) immunoblots relative to vehicle-treated samples. ***p <0.001,


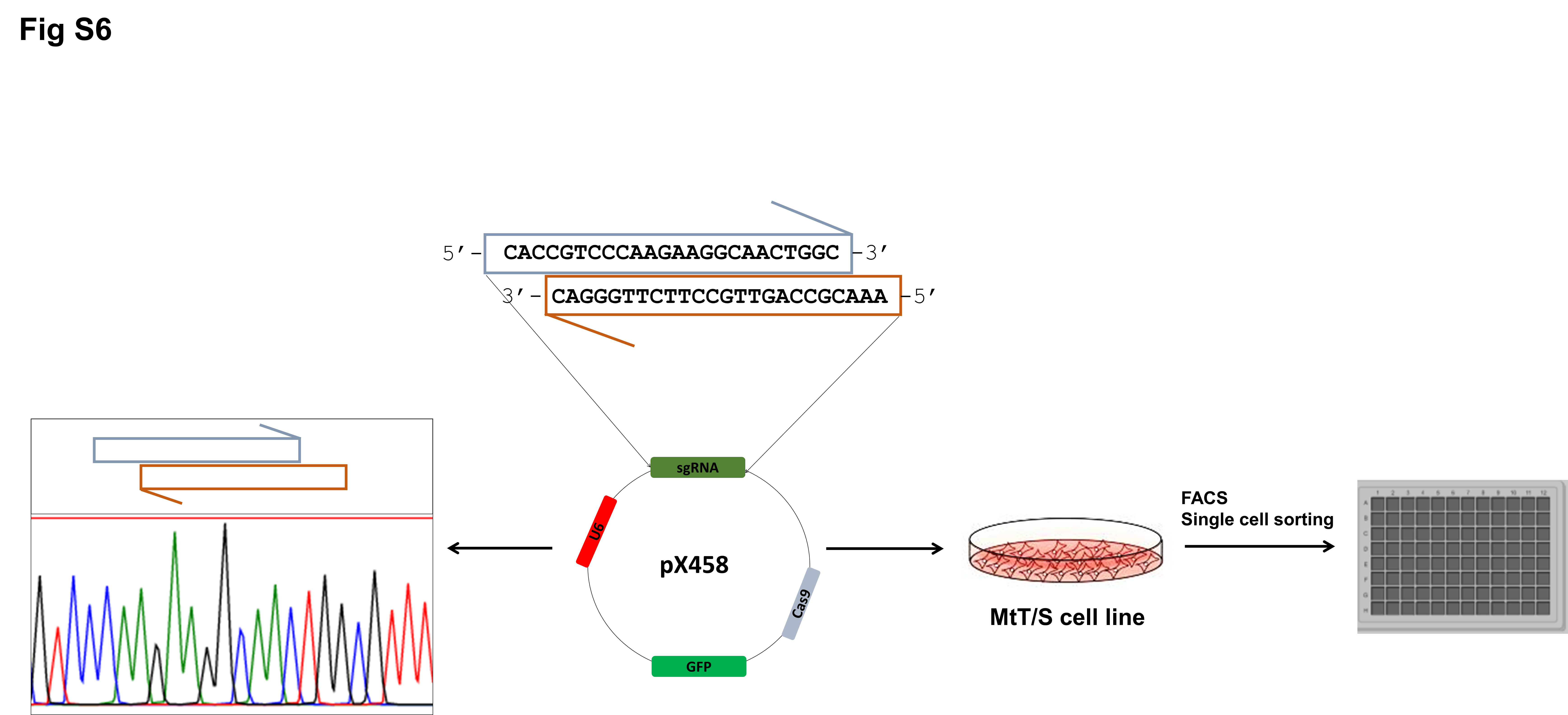


**FigS6- Experimental scheme of CRISPR/Cas9 induced knockout (KO) of MtT/S cell line.**

SgRNA was cloned into the plasmid pX458 (pSpCas9(BB)-2A-GFP) and verified by DNA sequencing. MtT/S cells were transfected with pX458 and after 48h, the cells were sorted. After three rounds of transfection-sorting, single cells were plated into 96-well plate.


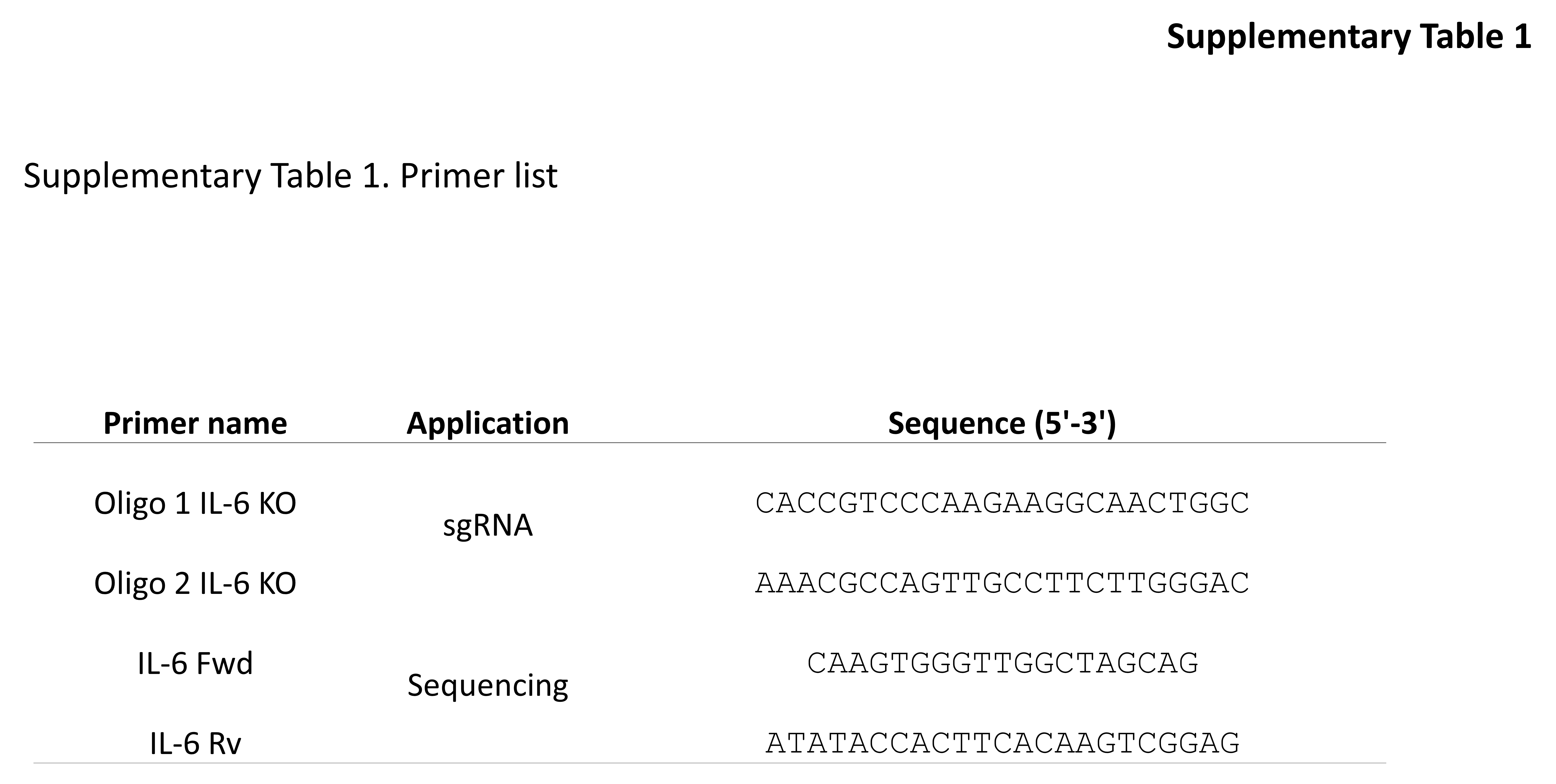

Supplement: Supplementary file 1 — Appendix S1. [file ACEL-23-e14258-s001.docx]
